# Effectiveness of behavior change techniques to address barriers to follow-up colonoscopy: results from an online survey and randomized factorial experiment

**DOI:** 10.1093/abm/kaae083

**Published:** 2024-12-31

**Authors:** Robert S Kerrison, Natalie Gil, Sandro Stoffel, Yasemin Hirst, Katriina L Whitaker, Colin Rees, Stephen Duffy, Christian von Wagner

**Affiliations:** School of Health Sciences, University of Surrey, Surrey, United Kingdom; School of Health Sciences, University of Surrey, Surrey, United Kingdom; Institute of Pharmaceutical Medicine, University of Basel, Basel, Switzerland; Department of Behavioural Science and Health, University College London, London, United Kingdom; Department of Behavioural Science and Health, University College London, London, United Kingdom; Applied Health Research Hub, University of Central Lancashire, Preston, United Kingdom; School of Health Sciences, University of Surrey, Surrey, United Kingdom; Faculty of Medical Sciences, Population Health Science Institute, Newcastle University, Newcastle, United Kingdom; Wolfson Institute of Population Health, Queen Mary University London, London, United Kingdom; Department of Behavioural Science and Health, University College London, London, United Kingdom

**Keywords:** barriers and facilitators, colorectal cancer screening, colonoscopy, online survey, online experiment, psycho-oncology

## Abstract

**Background:**

Nonattendance at colonoscopy is associated with reduced colorectal cancer (CRC) survival.

**Purpose:**

The aim of this research was to quantify barriers to colonoscopy and test the effectiveness of behavior change techniques (BCTs) to address them.

**Methods:**

Two studies were conducted. In the first study, participants were asked to imagine their next CRC screening result was abnormal, and were presented with the standard abnormal result letter used in the English CRC Screening Programme. Participants then completed a short survey. Multivariate regression tested associations between perceived barriers and intentions. In the second study, participants were randomly presented with a modified version of the abnormal results letter, which incorporated one or more BCTs, designed to target barriers identified in study 1, using a 2^8^ factorial design. Participants then completed the same survey used in study 1. Multivariate regression tested the effectiveness of the BCTs to modify target barriers and intentions.

**Results:**

In study 1, 5 items were associated with intentions, namely “Lack of understanding that CRC can be asymptomatic,” “Perceived importance of screening,” “Transport/travel,” “Shared decision making and family influenced participation,” and “Fear of pain and discomfort” (all *P*’s < .05). In study 2, the inclusion of a social support message, targeting “shared decision-making and family influenced participation,” facilitated independent decision making and increased intentions (both *P*’s < .05). There was no evidence to support the remaining 7 BCTs to modify barriers or intentions (all *P*’s < .05).

**Conclusions:**

Inclusion of a social support message facilitated independent decision-making and improved intentions.

## Teaser

Telling patients to discuss their colonoscopy invitation with their friends and family could improve attendance.

## Introduction

Colorectal cancer (CRC) is a leading cause of morbidity and mortality in the United Kingdom and North America.^1,2^ Several large randomized controlled trials (RCTs) have shown that regular fecal immunochemical test (FIT) screening, between the ages of 45 and 80, can significantly reduce CRC mortality through early detection.^3^ As a result, many healthcare providers now offer opportunistic or organized FIT-based screening to their patients.^4^

As with all screening, the extent to which the benefits of FIT are realized is highly dependent on the uptake of the test, as well as any follow-up investigations (colonoscopy being the gold standard for FIT-based screening).^5^ However, in a recent international survey of 35 FIT-based screening programs, Selby et al. found that the mean proportion of adults, with an abnormal FIT result, who attend colonoscopy, was 79%, with completion rates ranging from 39% in the program with the lowest attendance, to 100% in the country with the highest.^6^

In the United Kingdom, attendance at colonoscopy among those with an abnormal FIT-screening result (referred to as “follow-up colonoscopy”) is approximately 85%.^7^ Qualitative research has identified a number of barriers to follow-up colonoscopy, including psychological barriers (eg, fear of pain and discomfort), sociocultural barriers (eg, threat to masculinity), and practical barriers (eg, being unable to afford time off work).^8–11^ However, to date, no studies testing the associations between these barriers, and behavioral intentions, have been conducted, making it unclear which inhibit intentions (and, subsequently, behavior). Furthermore, few studies testing the effectiveness of behavioral interventions to address barriers/modify intentions for follow-up colonoscopy have been published, and none has reported positive results.^12,13^

The aim of this research, therefore, was to test associations between perceived barriers to colonoscopy and behavioral intentions, and to test the effectiveness of behavior change techniques (BCTs) to modify them.

## Methods: overview

### Design

Two studies were conducted. The first study was a close-ended online survey (study 1), which set out to test the association between perceived barriers (previously described in the qualitative literature)^8–11^ and behavioral intentions. The second study was an online factorial randomized experiment (study 2), which set out to test the effectiveness of embedding BCTs (targeting barriers identified in study 1) in the abnormal results letter used in the English CRC screening program. The decision to employ a factorial randomized design, over an RCT, was dictated by the findings of study 1, which identified multiple statistically significant barriers to behavioral intentions, and thereby necessitated the evaluation of multiple BCTs, which would have required many trial arms to test the effectiveness of each independently.

### Setting

Both studies were conducted in the United Kingdom, where FIT-based CRC screening, and follow-up colonoscopy, are free at the point of use (FIT screening is offered through the National Health Service, as part of an organized National Screening Programme, with invitations delivered biennially, between the ages of 54 and 74 in England, Northern Ireland, and Wales, and 50 and 74 in Scotland).

## Methods: study 1—online survey

### Study design

Study 1 comprised a hypothetical vignette, where participants were asked to imagine that their next FIT-screening result was abnormal. Participants were then presented with the standard abnormal results letter, which explains that further investigations are required, and that an appointment has been made for them to discuss their eligibility for further investigations with a nurse (this appointment is a pre-requisite for colonoscopy—patients who do not attend this appointment will not be offered a colonoscopy; therefore, intention to attend the nurse appointment is an important precedent for colonoscopy attendance). Following this, participants were asked to complete an online survey, which measured their intentions to attend the nurse appointment, how strongly they agreed with each of 29 statements (presented in a random order and designed to measure barriers to follow-up colonoscopy, identified in the UK qualitative literature—see “Measures” below)^8–11^ and their demographic characteristics.

### Participants

Participants were recruited (using convenience sampling) through Prolific: an online recruitment agency (co-founded by the University of Oxford) with access to over 50 000 adults living in the United Kingdom.^14^ Individuals registered with Prolific were potentially eligible to participate in the survey if they were aged 54-74 years of age (the screening age of patients in all countries, except Scotland, which includes those aged 50-53 years), and registered as living in the United Kingdom. Potentially eligible participants were contacted about the study by Prolific, who sent them a screening survey to complete (the screening survey was hosted online via Qualtrics: an online survey platform^15^). The screening survey asked participants whether they had ever taken part in CRC screening and, if so, whether they had ever received an abnormal screening result (and, therefore, been invited for colonoscopy). Those who had participated in CRC screening, and had not received an abnormal result, were subsequently invited to complete the online survey. These individuals were selected to increase the validity of the results (ie, ensure they were representative of those who take part in screening and were not influenced by previous experiences of follow-up colonoscopy within the program [a known confounder]).^8–11^ Participation was voluntary. Participants received up to £2.85 for taking part in the study (£0.50 for the screening survey and £2.35 for the full survey).

### Primary outcome

The primary outcome of interest was intention to attend the nurse appointment. This was assessed using the following item: “Based on the information in the letter you have just read, do you think you would go to the appointment with the nurse?” with a 5-point Likert scale including the following response options: “Yes, definitely,” “Yes, probably,” “Not sure,” “No, probably not,” and “No, definitely not.” For the purposes of the analysis, responses were dichotomized as “Yes, definitely” and “Any other response.” The rationale for this is that increased intention strength has been found to be a more accurate predictor of behavior and reduces the intention–behavior gap.^16^ For example, a previous study found that 82.2% of those who said they would “definitely” attend endoscopy if invited, went on to attend, compared with 59.9% of those who said they would “probably” attend.^17^ To minimize order effects, the order in which the response options were presented to participants was randomized.

### Measures: development of survey items

**Figure 1.**
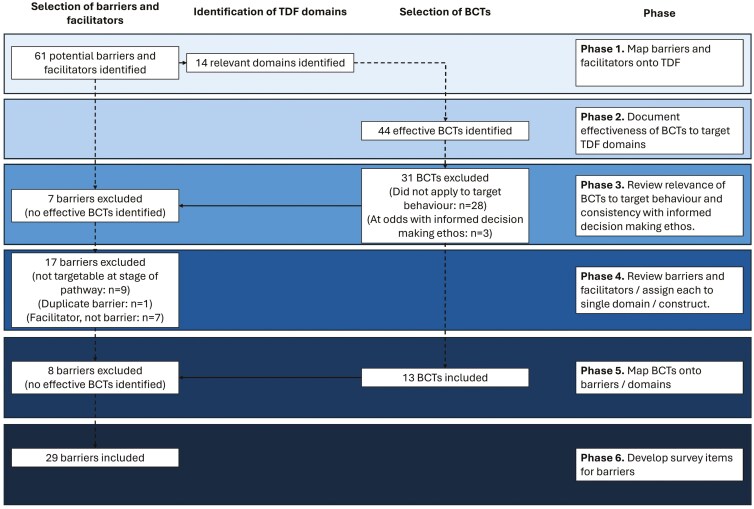
Visual representation of the barrier, domain, and BCT identification and selection process. Abbreviation: BCT, behavior change technique.

Twenty-nine items assessing barriers to colonoscopy attendance were developed from the UK qualitative literature,^8–11^ via a multiphase process. A visual representation of the process is presented in Figure 1.

In the first phase, 2 authors (R.K. and N.G.) independently mapped barriers and facilitators onto the Theoretical Domains Framework (TDF). The TDF is “an integrative framework developed from a synthesis of psychological theories as a vehicle to help apply theoretical approaches to interventions aimed at behavior change.”^18^ We chose this framework for 2 reasons. First, it allows researchers to identify candidate BCTs, using the BCT taxonomy (BCTT). Second, it was developed through a synthesis of existing frameworks, allowing a wider range of constructs to be included, compared with other available frameworks.

The authors’ decisions were combined in a matrix (Supplementary Table S1), to assess inter-rater agreement. Cells with a score of 0 indicated that neither author assigned the barrier/facilitator to the respective domain, while cells with a score of 1 indicated that 1 reviewer assigned the barrier/facilitator to the respective domain, and cells with a score of 2 indicated that both reviewers assigned the barrier/facilitator to the domain. Overall, inter-rater agreement was high (92.8%), with reviewers assigning barriers/facilitators to the same domain(s) in 755 of 812 instances (agree yes, *n* = 148; agree no, *n* = 607), and different decisions in 57. Discrepancies were resolved through discussion, during the fourth phase of the process (see below).

In the second phase, we created a matrix documenting the effectiveness of BCTs to target TDF domains, using data available from the BCTT^19^ (see Supplementary Table S2). The purpose of this was to identify candidate BCTs for inclusion in the online experiment (study 2) and to exclude barriers belonging to domains that were unlikely to be modifiable. In total, there was strong evidence (defined as evidence of an association in >50% of studies) to support the use of 44 BCTs (Supplementary Table S2). Those with no or mixed evidence (defined as no studies testing associations and evidence of an association in <50% of studies, respectively) were excluded (see Supplementary Table S3a and b, respectively).

In the third phase, we reviewed the 44 BCTs with strong evidence to support their use, and reflected on the descriptions of these BCTs, and their relevance to the target behavior (ie, attendance at a nurse appointment). An additional 31 BCTs were subsequently excluded, either because they did not apply to the target behavior (ie, they were related to other types of behavior, such as addiction), or were at odds with the informed decision-making ethos of the UK’s CRC screening programs (eg, they were considered to be coercive) (see Supplementary Table S3c and d, respectively).

After exclusions, 13 BCTs were potentially eligible for inclusion (Supplementary Table S4). None of the BCTs had evidence to support their use to target TDF domains assigned (by either reviewer) to 7 of the barriers and facilitators identified, and were excluded (Supplementary Table S3e).

In the fourth phase of the research, we revisited the barriers and facilitators, and allocated each of them to a specific construct, within a single domain (each TDF domain is made up of multiple constructs), to help identify which BCTs were best suited to targeting them (Supplementary Table S5). This was achieved through discussion between the 2 authors who completed phase 1 (R.K. and N.G.). Seventeen barriers and facilitators were subsequently excluded, on the basis that they either: (1) could not be targeted at this stage of the pathway (eg, “failed bowel prep”) (*n* = 9) (Supplementary Table S3f), (2) they were a duplicate of another barrier or facilitator (*n* = 1) (Supplementary Table S3g), or (3) they were a facilitator (*n* = 7) (Supplementary Table S3h), and thus could not be targeted.

In the fifth phase, we mapped BCTs onto the barriers, according to the TDF domain the barrier had been assigned, and whether the BCT was effective at targeting that domain (Supplementary Table S6). No BCTs targeting the agreed domains of 8 barriers could be identified, leading to the exclusion of these barriers from the survey (see Supplementary Table S3i). This led to the development of a matrix with 29 barriers that could be addressed by 13 BCTs (Supplementary Table S7).

In the sixth and final phase, once we had mapped the barriers onto the TDF and confirmed that those domains could be targeted by BCTs, we developed survey items to measure the barriers. This was achieved through iterative discussion between the authors and patient representatives (patient representatives were involved in the research, on the basis that involving patients can increase the effectiveness of translation later on^20^). Discussions continued until consensus about the wording of the items was achieved (see Supplementary Table S8 for an overview of the survey items used to measure the barriers). All of the survey items were expressed as statements and were measured using 5-point Likert scales, comprised of the following response options: “Strongly agree,” “Agree,” “Neither agree nor disagree,” “Disagree,” and “Strongly disagree.” To minimize order effects, the order in which the 29 survey items were presented to participants was randomized (but the order of response options was not). All items were included on a single page.

### Measures: demographic characteristics

Eight demographic characteristics were measured, including age (continuous), gender (categorized as man [including trans-man], or woman [including trans-woman], nonbinary and other [with the option to specify]), long-term conditions (yes or no), disabled (yes or no), mental health condition (yes or no), main language (English or any other language), ethnicity (White British/Irish or any other ethnicity), and educational attainment (ONC [Ordinary National Certificate]/BTEC [Business and Technology Education Council] or lower [≤Grade 10 equivalent] vs A-levels [Advanced-Levels], or higher [≥Grade 11 equivalent]) (see Supplementary Table S8). All were assessed via self-report. Demographic questions were presented on a separate, individual, page. Participants were unable to change responses after each page of the survey.

### Analysis

Data were collected between 12 and 16 January 2024. Descriptive statistics were used to describe intentions (frequencies and percentages), item scores (means and SDs), and sample characteristics (means, SDs, frequencies, and percentages).

Inferential statistics were used to test differences in demographic characteristics and mean item scores between “intenders” and “non-intenders.” In the first instance, univariate binary logistic regression was used, with a threshold probability value of .05 for statistical significance. Demographic characteristics and barrier items that were statistically significant in univariate models were entered as covariates in a multivariate model. The data were analyzed using SPSS (Statistical Software package for Social Statistics) (version 29.0). Odds ratios (ORs), adjusted ORs, and 95% CIs were reported.

### Missing data

Forced responses were used to minimize missing data. For demographic items, participants were given the option to respond “Prefer not to say.” These values were treated as missing, and the participants were excluded from the univariate and multivariate analyses (referred to as complete case analysis).

### Sample size calculation

The sample size calculation was designed to provide sufficient power (80%) and confidence (95%) to detect the smallest possible difference in item scores, between intenders and non-intenders, of 0.1 points (in either direction), assuming a standard variance of 0.5 points. This gave a sample size requirement of 785 participants. On the basis that 40% of invitees would not be eligible to participate in the study, we invited a total of 1448 participants. These assumptions were based on the results of pilot data, comprised of the first 200 respondents. Prolific IDs were used to ensure individuals only completed the survey once.

### Transparency

This study has been reported in accordance with the CHEcklist for Reporting Results of Internet E-Surveys (CHERRIES) guidelines (Supplementary Table S9).^21^

### Ethics

Participants were required to read an information sheet and consent form, which included information about the length of the survey, the principal investigators contact details, and a description of what data would be collected and how it would be stored (ie, on Qualtrics and later a secure University of Surrey server). Participants were unable to proceed with the survey until they confirmed they had read the information sheet and checked all 8 consent items. The study was approved by the University of Surrey (reference: FHMS23-24 060 EGA).

## Results: study 1—online survey

### Sample characteristics

**Figure 2.**
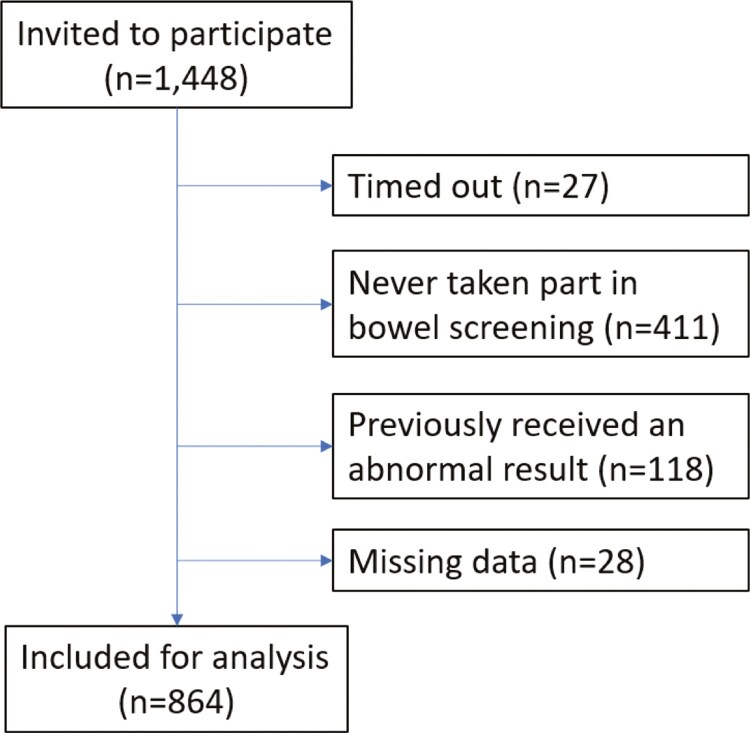
Flow of participants through study 1 (online survey).

One thousand four hundred and forty-eight adults were invited to complete the screening survey. Of these, 27 did not finish the survey, 411 had never participated in FIT screening, 118 had previously received an abnormal result, and 28 had missing data, leaving 864 eligible for inclusion (Figure 2). Of those eligible for inclusion, most were female (*n* = 483, 55.9%), did not have a long-term condition (*n* = 518, 60.0%), disability (*n* = 788, 91.2%), or mental health condition (*n* = 771, 89.2%), were of White British/Irish ethnicity (*n* = 816, 94.4%), spoke English as their main language (*n* = 857, 99.2%), and had an ONC/BTEC (Grade 11 equivalent) or higher qualification (*n* = 685, 79.3%) (see Table 1).

**Table 1.** Sample characteristics for study 1 (online survey) (*n* = 864).

Intentions	
Yes, definitely	751 (86.9)
Any other response	113 (13.1)
Age (mean)	
Continuous (y)	—
Gender	
Male	380 (44.0)
Female	483 (55.9)
Non-binary	1 (0.1)
Long-term conditions	
No	518 (60.0)
Yes	346 (40.0)
Disability	
No	788 (91.2)
Yes	76 (8.8)
Mental health	
No	771 (89.2)
Yes	93 (10.8)
Main language	
English	857 (99.2)
Any other language	9 (0.8)
Ethnicity	
White British/Irish	816 (94.4)
Any other ethnic group	48 (5.6)
Education	
≤O-level or GCSE Grades A–C	179 (20.7)
≥ONC/BTEC	685 (79.3)

### Intentions: all participants

Overall, intentions to attend the nurse appointment were high (86.9%, *n* = 751) indicating that they would definitely attend the appointment.

### Univariate analyses: all participants

In the univariate analyses, all 29 barrier items were significantly associated with intentions to attend the nurse appointment (all *P*’s < .05; see Table 2). Of the demographic items, only mental health and education were significantly associated with intentions (both *P*’s < .05), with individuals self-reporting a mental health condition being less likely to say they would definitely attend, and individuals with an ONC/BTEC (Grade 11) or higher qualification being more likely to say they would definitely attend.

**Table 2.** Predictors of colonoscopy intentions: mean scores, proportions, and unadjusted and adjusted odds ratios—results from the univariate and multivariate binary logistic regression analysis (*n* = 864).

	Mean item scores for respondents answering Yes probably, Not sure, No probably not, and No definitely not (range 1-5; 1 = Strongly disagree, 5 = Strongly agree)	Mean item scores for respondents answering Yes definitely (range 1-5; 1 = Strongly disagree, 5 = Strongly agree)	OR (95% CI)	aOR (95% CI)
Psychological variables (*R*^2^ = 0.44)				
1.1. Knowledge (knowledge of condition/scientific rationale)				
3.2.1. Lack of understanding that bowel cancer can be asymptomatic and the test is looking for invisible traces of blood (Bowel cancer screening is only useful for people with symptoms)	1.91	1.33	0.39 (0.30-0.50)***	0.68 (0.47-0.97)*
3.5.4.2. Belief that cancer is a treatable disease (Bowel cancer is not a treatable disease)	2.02	1.61	0.51 (0.39-0.65)***	0.91 (0.58-1.43)
1.2. Knowledge (procedural knowledge)				
1.3.1. Hearing other people’s experiences with colonoscopy (Other people’s experiences with colonoscopy have put me off going for colonoscopy myself)	2.61	1.81	0.46 (0.38-0.56)***	1.00 (0.73-1.37)
3.2.2. Lack of awareness and understanding of colonoscopy procedure (I have a good understanding of what having a colonoscopy would involve)	2.44	2.15	0.75 (0.62-0.91)**	0.97 (0.73-1.37)
2.3. Beliefs about capabilities (self-efficacy)				
4.2.2. Existing health conditions interfering with the ability to complete procedure or bowel prep (I have existing health conditions that would prevent me from being able to have a colonoscopy)	1.54	1.27	0.48 (0.36-0.65)***	1.52 (0.92-2.51)
3.1. Beliefs about consequences (beliefs)				
1.2.4. Fatalistic beliefs (Being diagnosed with bowel cancer is a death sentence)	2.46	2.06	0.59 (0.47-0.74)***	1.22 (0.83-1.79)
1.2.7. Lack of trust in Western Medicine (Western medicine is not effective at treating bowel cancer)	2.21	1.61	0.45 (0.35-0.56)***	0.77 (0.53-1.10)
3.5.6. Perceived importance of screening (It is important to take part in bowel cancer screening)	1.87	1.16	0.20 (0.15-0.29)***	0.35 (0.24-0.50)***
3.9.6. The role of God in determining the future (It is God’s decision who lives and dies, medicine cannot change that)	1.64	1.28	0.59 (0.47-0.73)***	1.02 (0.71-1.47)
5.2. Environmental context and resources (resources/material resources)				
2.1.1. Language barriers (I would need an interpreter to translate during the nurse appointment)	1.23	1.10	0.53 (0.35-0.79)**	1.57 (0.73-3.40)
5.6. Environmental context and resources (barriers and facilitators)				
2.2.1. Transport/travel (I would find it difficult to travel to the appointment with the nurse)	2.36	1.54	0.41 (0.33-0.50)***	0.57 (0.41-0.79)***
2.2.4. Lack of car parking (If I drove to the appointment, it would be difficult to find a car parking space at my local hospital)	3.65	3.26	0.75 (0.63-0.90)**	0.96 (0.76-1.22)
2.2.5. Indirect costs (Going to the appointment (eg, parking, public transport, time off work, etc.) would cost me a lot of money)	2.51	2.03	0.65 (0.54-0.78)***	0.99 (0.75-1.30)
7.2. Social influences (social norms)				
1.2.2. Colonoscopy, colon and rectum “culturally taboo” topics (I would not be able to discuss this appointment with my friends and family, as this type of thing is very sensitive)	2.47	1.83	0.55 (0.46-0.66)***	1.02 (0.77-1.35)
1.2.5. Unable to accept blood products (I would be worried that a blood transfusion might be needed if I had a colonoscopy, and I am unwilling to have these on religious grounds)	1.38	1.14	0.43 (0.30-0.61)***	0.98 (0.58-1.65)
7.5. Social influences (group norms)				
1.2.3. Gender and engagement with healthcare (It is fine for women to have colonoscopy, but not men)	1.46	1.22	0.30 (0.21-0.43)***	0.81 (0.45-1.44)
7.6. Social influences (social support) 0.70				
1.4.1. Reliance on family and friends as unofficial interpreters (I would need to take a friend or family member with me to translate what the nurse was saying)	1.44	1.13	0.59 (0.46-0.77)***	0.76 (0.49-1.18)
1.4.2. Reliance on family for travel and transport (I would need a friend or family member to take me to the nurse appointment)	2.33	1.94	0.76 (0.65-0.89)***	1.12 (0.81-1.56)
1.4.3. Reliance on family for emotional support (I would need to take a friend or family member with me to the nurse appointment for emotional support)	2.67	2.35	0.82 (0.70-0.95)**	1.07 (0.80-1.43)
3.9.4. Shared decision-making and family-influenced participation (I would only go to the nurse appointment if a friend or family member agreed it was in my best interest to attend)	2.04	1.40	0.44 (0.36-0.55)***	0.68 (0.47-0.99)*
7.7. Social influences (power)				
3.9.3. Reliance on medical professional/authority (I would only go to the nurse appointment if my general practitioner told me I should go)	2.19	1.44	0.39 (0.32-0.49)***	0.76 (0.54-1.06)
8.1. Emotion (fear) 0.81				
3.1.2. Fear of pain and discomfort (I would be worried that the colonoscopy would be painful or uncomfortable)	4.14	3.51	0.45 (0.34-0.59)***	0.63 (0.40-0.98)*
3.5.4.1. Fear of cancer (I would be scared that the colonoscopy would find cancer)	3.86	2.93	0.79 (0.63-1.00)*	1.14 (0.80-1.64)
8.2. Emotion (anxiety)				
3.1.1. Concerns about doing the bowel preparation (I would be worried about doing the bowel preparation/drinking powerful laxatives before the colonoscopy)	3.91	3.72	0.55 (0.46-0.66)***	0.81 (0.61-1.06)
3.1.3. Concerns about test invasiveness (I am worried about the invasive nature of colonoscopy)	3.66	2.80	0.48 (0.39-0.59)***	0.79 (0.56-1.13)
3.1.4. Shame and embarrassment (Going for colonoscopy would be shameful and/or embarrassing)	2.66	1.80	0.55 (0.47-0.65)***	0.81 (0.63-1.04)
3.3.1. Anxiety (I would feel anxious about going to the nurse appointment)	3.91	3.40	0.63 (0.51-0.78)***	1.08 (0.78-1.50)
3.3.3. Avoidance (I would prefer not to know whether I had bowel cancer)	2.00	1.31	0.41 (0.33-0.52)***	0.87 (0.61-1.23)
Demographic variables (*R*^2^ = 0.47)				
Age				
Years (continuous)	—	—	0.99 (0.95-1.03)	—
Gender				
Male	46 (12.1)	334 (87.9)	1.00	—
Female	67 (13.9)	416 (86.1)	0.86 (0.57-1.28)	—
Long-term conditions				
No	71 (13.7)	447 (86.3)	1.00	—
Yes	42 (12.1)	304 (87.9)	1.15 (0.76-1.73)	—
Disability				
No	101 (12.8)	687 (87.2)	1.00	—
Yes	12 (15.8)	64 (84.2)	0.78 (0.41-1.50)	—
Mental health				
No	93 (12.1)	678 (87.9)	1.00	1.00
Yes	20 (21.5)	73 (78.5)	0.50 (0.29-0.86)*	0.72 (0.32-1.61)
Main language				
English	111 (13.0)	746 (87.0)	1.00	—
Other	2 (28.6)	5 (71.4)	0.37 (0.07-1.94)	—
Ethnicity				
White British/Irish	105 (12.9)	711 (87.1)	1.00	—
Any Other Ethnicity	8 (16.7)	40 (83.3)	0.74 (0.34-1.62)	—
Education				
≤O-level or GCSE Grades A–C (≤Grade 10)	44 (24.6)	135 (75.4)	1.00	1.00
≥ONC/BTEC (≥Grade 11)	69 (10.1)	616 (89.9)	2.91 (1.91-4.44)***	2.78 (1.57-4.94)***

Abbreviations: aOR, adjusted odds ratio; BTEC, Business and Technology Education Council; ONC, Ordinary National Certificate; OR, odds ratio. *p<0.05 **p<0.01***p<0.001. Odds ratios and confidence intervals in bold indicates results are statistically significant.

### Multivariate analysis: all participants

In the multivariate analysis, higher agreement scores for the following 5 items were associated with reduced intentions to attend the nurse appointment: (1) “Lack of understanding that bowel cancer can be asymptomatic,” (2) “Perceived importance of screening,” (3) “Transport/travel,” (4) “Shared decision making,” and (5) “Fear of pain and discomfort” (all *P*’s < .05; see Table 2).

Of the demographic items included in the multivariate analysis, only education remained significantly associated with intentions, with individuals with an ONC/BTEC (Grade 11) or higher qualification being more likely to say they would definitely attend (*P* < .001).

### Univariate analyses: any other ethnic group

As many of the barriers identified in the qualitative studies were specific to ethnic minority groups (eg, “Lack of trust in western medicine”),^8–11^ we conducted a subgroup analysis with participants of any other ethnic group. Eight items in the subgroup analysis were associated with intentions (all subgroup analyses were univariate, due to sample size limitations). Three were previously identified as predictors in the multivariate analysis conducted with all participants (ie, “Perceived importance of screening,” “Shared decision making, and family influenced participation,” and “Transport/travel”), and 5 were not (namely: “Lack of trust in western medicine,” “Cultural taboos,” “Reliance on medical professional/authority,” “Shame and embarrassment,” and “Avoidance” (all *P*’s < .05; see Supplementary Table S10).

## Methods: study 2—online experiment

### Study design

Study 2 was a randomized factorial online experiment. Participants were presented with the same hypothetical vignette as study 1, and were asked to imagine that their next CRC screening result was abnormal. Unlike study 1, however, participants were randomly presented with a modified version of the abnormal results letter, rather than the standard abnormal results letter. The modified letter contained the same wording as the standard letter, but included up to 8 additional statements, each encompassing a BCT designed to target a specific barrier identified in study 1 (see “Selection of BCTs and development of statements” section). The same survey used in study 1 was then presented to participants to measure their intentions, barriers, and demographics.

### Participants

As with study 1, participants were recruited through Prolific, using the same criteria and screening survey. To minimize potential confounding from previous participation, participants were not eligible for inclusion if they had participated in study 1.

### Selection of BCTs and development of statements

In study 1, we identified 10 unique barriers that were significantly associated with intentions: 5 in the multivariate analysis with all participants, and 5 in the univariate analyses with those from ethnic minority groups. We (the researchers and patient representatives) reviewed these barriers and, through discussion, selected 8 as targets for the online experiment (“bowel cancer screening is only useful for people with symptoms” and “it is important to take part in bowel cancer screening” were excluded, as they related more to completing the screening test).

Having previously mapped the barriers onto the TDF and identified effective BCTs to target relevant domains, using the BCTT, we then developed targeted messages to address barriers, incorporating the relevant BCTs. To do this, we selected a single BCT to target each barrier, and drafted an initial message consistent with the definition of the BCT. Messages were then refined through iterative discussion between authors and members of the public, until agreement was achieved. An overview of the targeted barriers, relevant domains, selected BCTs, and message wording is provided in Table 3.

**Table 3.** Overview of selected barriers and corresponding survey items.

Barrier	TDF domain	BCT	Message
Avoidance	Emotion (anxiety)	BCT 1. Reduce negative emotions	If bowel cancer is found, it is usually treatable. It is best to find out as soon as possible.
Lack of trust in Western Medicine	Beliefs about consequences (beliefs)	BCT 2. Information about health consequences	Advances in modern medicine mean that most people, diagnosed through screening, survive 10 or more years.
Shared decision-making and family-influenced participation	Social influences (social support)	BCT 3. Social support (unspecified)	If you are unsure whether you should attend the consultation, you can discuss this letter with your friends and family.
Colonoscopy, colon, and rectum “culturally taboo” topics	Social influences (social norms)	BCT 4. Social support (practical)	We understand talking about colonoscopy can be difficult. If you would like support discussing it with your loved ones, please contact us.
Shame and embarrassment	Emotion (anxiety)	BCT 5. Reduce negative emotions	Most people do not find colonoscopy embarrassing, and reasonable adjustments are made to protect your modesty. A gown will be provided to cover your body.
Fear of pain and discomfort	Emotion (fear)	BCT 6. Reduce negative emotions	If you are worried the test might be uncomfortable, we can arrange sedation to help relax you.
Transport/travel	Environmental context and resources (barriers and facilitators)	BCT 7. Social support (practical)	If you need help traveling to and from the consultation, please call us to arrange free transport.
Reliance on medical professional/authority	Social Influences (power)	BCT 8. Information about others’ approval	We have told your GP that we have offered you a screening practitioner appointment. They recommend you attend this appointment.

Abbreviations: BCT, behavior change technique; TDF, Theoretical Domains Framework.

*p<0.05 Odds ratios and confidence intervals in bold indicates results are statistically significant.

### Randomization and blinding

Participants were randomly presented with 1 of the 256 variations of the abnormal results letter (each containing a combination of one or more of the messages), via Qualtrics inhouse random allocation software, using a 2^8^ randomized factorial design. As the intervention was overt, it was not possible to blind participants to the intervention. The researchers were blinded to allocation, up until receipt and analysis of data.

### Primary outcome

The primary outcome of interest was intention to attend the nurse appointment, which was assessed using the same item and response options as study 1.

### Measures

Agreement with each of the 29 items was measured as described in study 1. Demographic characteristics were also assessed using the same items and response options.

### Analysis

Data were collected between April 29 and May 1, 2024. Descriptive statistics were used to describe sample characteristics (means, SDs, frequencies, and percentages, accordingly). Descriptive statistics were also used to describe intentions/targets (frequencies and percentages, means and SDs, respectively) by BCT exposure. To achieve this, dummy variables were generated for each BCT, with a value of 0 indicating that the message was not present, and a value of 1 indicating that the message was present, alone or in combination with one or more other messages.

Inferential statistics were used to test the effectiveness of the BCTs to modify respective targets and intentions to attend the nurse appointment. In the first instance, univariate binary logistic regression was used, with a threshold probability value of .05 used to determine statistical significance. BCTs and demographic characteristics that were associated with intentions or target constructs in the univariate models were entered into a multivariate model.

### Missing data

As with study 1, forced responses and complete case analysis were used.

### Sample size calculations

The sample size calculation was designed to provide sufficient power (80%) and confidence (95%) to detect the smallest possible difference in intentions, between BCT groups, of 5 percentage-points. A baseline intention of 87% was assumed, based on the results of the online survey. This gave a sample size requirement of 589 participants, per trial arm (1178, total). On the basis that 40%-50% of invitees would be ineligible (as seen with the online survey), we invited twice the number required (2436 total).

### Transparency

This study has been reported in accordance with the Reporting of Factorial Randomized Trials Extension of the CONSORT 2010 Statement guidelines (Supplementary Table S11).^22^

### Ethics

The ethical procedures and approvals were the same as those described in study 1.

## Results: study 2—online experiment

### Sample characteristics

**Figure 3.**
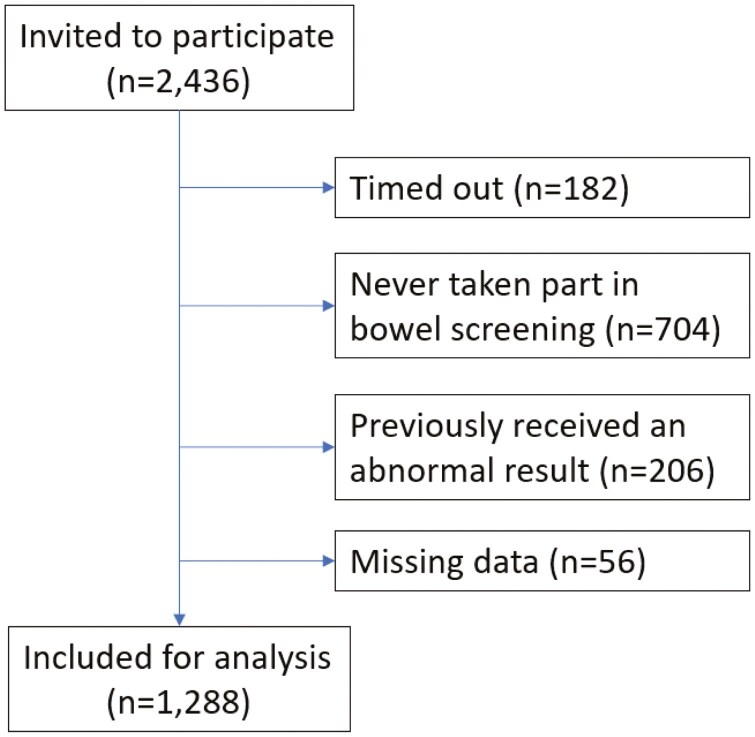
Flow of participants through study 2 (online experiment).

Two thousand four hundred and thirty-six adults were invited to complete the screening survey. Of these, 182 did not finish the survey, 704 had never taken part in bowel screening, 206 had previously received an abnormal result, and 56 had missing data, leaving 1288 eligible for inclusion (see Figure 3). Of those eligible for inclusion, most were female (*n* = 790, 61.3%), did not have a long-term condition (*n* = 752, 58.3%), disability (*n* = 1151, 89.3%), or mental health condition (*n* = 1138, 88.4%), were of White British/Irish ethnicity (*n* = 1211, 94.0%), spoke English as their main language (*n* = 1271, 98.7%), and had an ONC/BTEC (Grade 11) or higher (*n* = 998, 77.5%) (Table 4).

**Table 4.** Sample characteristics for study 2 (online experiment) (*n* = 1288).

Intentions	
Yes, definitely	1115 (86.6)
Any other response	173 (13.4)
Age (mean, SD)	
Continuous (y)	62.20 (5.11)
Gender	
Male	499 (38.7)
Female	790 (61.3)
Long-term conditions	
No	752 (58.3)
Yes	537 (41.7)
Disability	
No	1151 (89.3)
Yes	138 (10.7)
Mental health	
No	1138 (88.4)
Yes	150 (11.6)
Main language	
English	1271 (98.7)
Any other language	17 (1.3)
Ethnicity	
White British/Irish	1211 (94.0)
Any other ethnic group	77 (6.0)
Education	
≤O-level or GCSE Grades A–C (≤Grade 10)	290 (22.5)
≥ONC/BTEC (≥Grade 11)	998 (77.5)

Abbreviations: BTEC, Business and Technology Education Council; ONC, Ordinary National Certificate.

### Intentions

Overall, intentions to attend the nurse appointment were high (86.6%, *n* = 1115) indicating that they would definitely attend the appointment.

### Univariate analysis

In the univariate analyses, only the social support message (BCT 3), alone or in combination with other messages, was effective at modifying intentions (see Table 5). Of the demographic characteristics, increasing age was associated with increased intentions, as was having a long-term condition.

**Table 5.** Effectiveness of BCTs to modify intentions: number, proportions, and unadjusted and adjusted odds ratios—results from the univariate and multivariate binary logistic regression analyses (all participants) (*n* = 1288).

	All other responses *n* (%)	Yes, definitely *n* (%)	OR (95% CI)	aOR (95% CI)
BCTs				
BCT 1. Reduce negative emotions (off) (*n* = 646)	96 (14.9)	550 (85.1)	1.00	—
BCT 1. Reduce negative emotions (on) (*n* = 642)	77 (12.0)	565 (88.0)	1.28 (0.93-1.77)	—
BCT 2. Information about health consequences (off) (*n* = 652)	99 (15.2)	553 (84.8)	1.00	—
BCT 2. Information about health consequences (on) (*n* = 636)	74 (11.6)	562 (88.4)	1.36 (0.98-1.88)	—
BCT 3. Social support (unspecified) (off) (*n* = 641)	101 (15.8)	540 (84.2)	1.00	1.00
BCT 3. Social support (unspecified) (on) (*n* = 647)	72 (11.1)	575 (88.9)	1.49 (1.08-2.07)*	1.47 (1.06-2.04)*
BCT 4. Social support (practical) (off) (*n* = 649)	90 (13.9)	559 (86.1)	1.00	—
BCT 4. Social support (practical) (on) (*n* = 639)	83 (13.0)	556 (87.0)	1.08 (0.78-1.49)	—
BCT 5. Reduce negative emotions (off) (*n* = 651)	85 (13.1)	566 (86.9)	1.00	—
BCT 5. Reduce negative emotions (on) (*n* = 637)	88 (13.8)	549 (86.2)	0.94 (0.68-1.29)	—
BCT 6. Reduce negative emotions (off) (*n* = 667)	95 (14.2)	572 (85.8)	1.00	—
BCT 6. Reduce negative emotions (on) (*n* = 621)	78 (12.6)	543 (87.4)	1.16 (0.84-1.60)	—
BCT 7. Social support (practical) (off) (*n* = 645)	81 (12.6)	564 (87.4)	1.00	—
BCT 7. Social support (practical) (on) (*n* = 643)	92 (14.3)	551 (85.7)	0.86 (0.62-1.19)	—
BCT 8. Information about others approval (off) (*n* = 664)	100 (15.1)	564 (84.9)	1.00	—
BCT 8. Information about others approval (on) (*n* = 624)	73 (11.7)	551 (88.3)	1.34 (0.97-1.85)	—
Age				
Years (continuous)	—	—	1.04 (1.00-1.07)*	1.03 (1.00-1.07)
Gender				
Male	46 (12.1)	334 (87.9)	1.00	—
Female	67 (13.9)	416 (86.1)	0.84 (0.60-1.18)	—
Long-term conditions				
No	71 (13.7)	447 (86.3)	1.00	1.00
Yes	42 (12.1)	304 (87.9)	1.41 (1.01-1.97)*	1.35 (0.97-1.89)
Disability				
No	101 (12.8)	687 (87.9)	1.00	—
Yes	12 (15.8)	64 (84.2)	1.11 (0.65-1.91)	—
Mental health				
No	93 (12.1)	678 (87.9)	1.00	—
Yes	20 (21.5)	73 (78.5)	0.79 (0.50-1.27)	—
Main language				
English	111 (13.0)	746 (87.0)	1.00	—
Other	2 (28.6)	5 (71.4)	0.50 (0.16-1.55)	—
Ethnicity				
White British/Irish	105 (12.9)	711 (87.1)	1.00	—
Any Other Ethnicity	8 (16.7)	40 (83.3)	0.57 (0.32-1.01)	—
Education				
≤O-level or GCSE Grades A–C (≤Grade 10)	44 (15.2)	246 (84.8)	1.00	—
≥ONC/BTEC (≥Grade 11)	129 (12.9)	869 (87.1)	1.21 (0.83-1.75)	—

Abbreviations: aOR, adjusted odds ratio; BCT, behavior change technique; BTEC, Business and Technology Education Council; ONC, Ordinary National Certificate; OR, odds ratio. *p<0.05 Odds ratios and confidence intervals in bold indicates results are statistically significant.

Social support (BCT 3) was also effective at modifying the domain it was intended to address (ie, Social influence), while the other BCTs were not (see Table 6).

**Table 6.** Effectiveness of BCTs to modify target constructs: mean scores and unadjusted and adjusted odds ratios—results from the univariate and multivariate binary logistic regression analyses (*n* = 1288).

	Avoidance		Lack of trust in Western Medicine		Shared decision making and family influenced participation		Colonoscopy, colon, and rectum “culturally taboo” topics		Shame and embarrassment		Fear of pain and discomfort		Transport/travel		Reliance on medical professional/authority	
	Mean (SD)	*t* (*P*)	Mean (SD)	*t* (*P*)	Mean (SD)	*t* (*P*)	Mean (SD)	*t* (*P*)	Mean (SD)	*t* (*P*)	Mean (SD)	*t* (*P*)	Mean (SD)	*t* (*P*)	Mean (SD)	*t* (*P*)
BCT 1 Reduce negative emotions (off) (*n* = 646)	1.43 (0.82)	0.04 (.965)	—	—	—	—	—	—	—	—	—	—	—	—	—	—
BCT 1 Reduce negative emotions (on) (*n* = 642)	1.43 (0.79)		—	—	—	—	—	—	—	—	—	—	—	—	—	—
BCT 2 Information about health consequences (off) (*n* = 652)	—	—	1.65 (.76)	0.204 (.838)	—	—	—	—	—	—	—	—	—	—	—	—
BCT 2 Information about health consequences (on) (*n* = 636)	—	—	1.64 (.79)		—	—	—	—	—	—	—	—	—	—	—	—
BCT 3 Social support (unspecified) (off) (*n* = 641)	—	—	—	—	1.48 (.68)	**−2.047** **(.041)**	—	—	—	—	—	—	—	—	—	—
BCT 3 Social support (unspecified) (on) (*n* = 647)	—	—	—	—	1.56 (.83)		—	—	—	—	—	—	—	—	—	—
BCT 4 Social support (practical) (off) (*n* = 649)	—	—	—	—	—	—	1.89 (.94)	1.104 (.270)	—	—	—	—	—	—	—	—
BCT 4 Social support (practical) (on) (*n* = 639)	—	—	—	—	—	—	1.84 (.90)		—	—	—	—	—	—	—	—
BCT 5 Reduce negative emotions (off) (*n* = 651)	—	—	—	—	—	—	—	—	1.98 (1.162)	−0.246 (.806)	—	—	—	—	—	—
BCT 5 Reduce negative emotions (on) (*n* = 637)	—	—	—	—	—	—	—	—	2.00 (1.076)		—	—	—	—	—	—
BCT 6 Reduce negative emotions (off) (*n* = 667)	—	—	—	—	—	—	—	—	—	—	3.62 (1.047)	1.255 (.210)	—	—	—	—
BCT 6 Reduce negative emotions (on) (*n* = 621)	—	—	—	—	—	—	—	—	—	—	3.55 (1.085)		—	—	—	—
BCT 7 Social support (practical) (off) (*n* = 645)	—	—	—	—	—	—	—	—	—	—	—	—	2.17 (1.229)	1.041 (.298)	—	—
BCT 7 Social support (practical) (on) (*n* = 643)	—	—	—	—	—	—	—	—	—	—	—	—	2.10 (1.219)		—	—
BCT 8 Information about others approval (off) (*n* = 664)	—	—	—	—	—	—	—	—	—	—	—	—	—	—	1.60 (.809)	—0.444 (.657)
BCT 8 Information about others approval (on) (*n* = 624)	—	—	—	—	—	—	—	—	—	—	—	—	—	—	1.62 (.864)	

### Multivariate analysis

In the multivariate analysis, social support (BCT 3) remained significantly associated with increased intentions to attend the nurse appointment (*P* < .05), but age and having a long-term condition did not (both *P*’s > .05).

### Subgroup analysis

A subgroup analysis was conducted to test whether BCTs were effective at addressing intentions (Supplementary Table S12) and barriers (Supplementary Table S13) in ethnic minority groups. No statistically significant effects were observed (all *P*s > .05).

### Interaction analyses

An interaction analysis was conducted to test for synergistic and antagonistic relationships between statements sharing the same BCT concept. The only combination that was found to be effective at modifying intentions was a combination of social support messages (BCTs 3 and 4; see Supplementary Table S14). There was no evidence (*P* > .05) that these BCTs behaved synergistically to address their respective targets (see Supplementary Table S15). There was evidence, however, that messages that employed the BCT “Reduce negative emotion messages” (BCTs 5 and 6) behaved synergistically to reduce fear of pain and discomfort (*P* < .05; Supplementary Table S15), but not intentions (*P* > .05; Supplementary Table S14).

## Discussion

### Summary of main findings

This study provides novel insights into the barriers to colonoscopy intentions, within UK national CRC screening programs, and the effectiveness of BCTs to address them. First, it indicates that “Lack of understanding that bowel cancer can be asymptomatic” (Knowledge [Knowledge of condition/scientific rationale]), “Perceived importance of screening” (Beliefs about consequences [Beliefs]), “Transport/travel” (Environmental context and resources [Barriers and facilitators]), “Shared decision making, and family influenced participation” (Social influences [Social support]), and “Fear of pain and discomfort” (Emotion [fear]) are associated with intentions, within the general population. Second, it indicates that “Lack of trust in Western Medicine” (Beliefs about consequences [Beliefs]), “Colonoscopy, colon and rectum as ‘culturally taboo’ topics” (Social influences [Social norms]), “Reliance on medical professional/authority” (Social influences [Power]), “Shame and embarrassment” (Emotion [Anxiety]), and “Avoidance” (Emotion [Anxiety]) are associated with intentions among ethnic minority groups, specifically. Third, it indicates that the inclusion of a social support message, within the abnormal results letter, modifies social influence, and, in turn, intentions.

### Comparisons with existing literature

The results of this study share several consistencies with the extant literature. First, the finding that, in the univariate analyses, all of the barriers identified in previous qualitative studies are associated with intentions, supports previous subjective interpretations of the barriers to colonoscopy.^8–11^ The present study builds on these findings, by disentangling which are the most pertinent in influencing intentions, through the use of a multivariate model, which highlights that “Lack of understanding that bowel cancer can be asymptomatic,” “Perceived importance of screening,” “Transport/travel,” “Shared decision making and family influenced participation,” and “Fear of pain and discomfort” are the most important for the general population. Second, the finding that “Lack of trust in Western Medicine,” “Colonoscopy, colon and rectum as ‘culturally taboo’ topics,” “Reliance on medical professional/authority,” “Shame and embarrassment,” and “Avoidance” are associated with intentions among ethnic minority groups, specifically, is consistent with previous qualitative research exploring ethnic differences in barriers to colonoscopy.^11^

The results of this study are also consistent with the findings of other experimental studies. For example, the finding that BCTs are not effective at modifying emotional barriers, such as fear, when embedded within a letter, has previously been documented in relation to colonoscopy by Travis et al., who found similar observations when attempting to reduce anxiety.^12,13^ Taken together, these studies suggest that researchers should consider alternative approaches to reducing emotional barriers, such as patient navigation, which allows greater discussion and targeted advice,^23,24^ as small changes to the letter are not sufficient.

### Policy implications and future research

The results of this study suggest that interventions seeking to improve uptake in the general population should target “Lack of understanding that bowel cancer can be asymptomatic and the test is looking for invisible traces of blood,” “Perceived importance of screening,” “Transport/travel,” “Shared decision making and family influenced participation,” and “Fear of pain and discomfort,” while interventions seeking to reduce ethnic inequalities should target “Lack of trust in Western Medicine,” “Colonoscopy, colon and rectum ‘culturally taboo’ topics,” “Reliance on medical professional/authority,” “Shame and embarrassment,” and “Avoidance.”

The results of this study also support the inclusion of a social support message, within the abnormal results letter, to modify social influence as a barrier to the nurse appointment, and intentions to attend. Further research is needed, however, to test whether improvement in intentions translates to an improvement in attendance^25^; eg, a “real-world” RCT.

Finally, exploratory analyses, testing whether BCTs were effective at targeting barriers and intentions for ethnic minority groups, specifically, found no evidence to support their use. This is likely to be a power issue, as only a small proportion of individuals were of non-White British ethnicity. Further studies, with more ethnically diverse panels, are required to confirm whether these interventions might be effective for these groups. Alternative strategies, such as patient navigation, which allow cultural barriers to be elicited and addressed, might particularly be worth considering.^23,24^

### Strengths and limitations

This study has a number of strengths. First, it used a randomized factorial design, which is an efficient design enabling the testing of multiple interventions, without increasing the overall sample size, enabling us to test the effectiveness of multiple BCTs simultaneously. Second, it measured a number of constructs, other than intentions, allowing us to confirm the mechanisms by which BCTs modify intentions, rather than simply confirm whether they are effective. Finally, the randomization of participants, within the study design, minimized confounding from external factors, such as selection bias.

This study also has a number of important limitations. First, it measured intentions, rather than behavior, and it is well documented that behaviors can differ substantially.^26,27^ Second, it tested the effectiveness of BCTs in a hypothetical scenario, and not an ecological one. As such, the study lacks ecological validity and is subject to hypothetical bias. Finally, the samples in both the survey and online experiment were not representative of the general population. A disproportionate number of participants were of White British ethnicity/nationality and had high educational attainment,^28^ reducing generalizability.

## Supplementary material

Supplementary material is available at *Annals of Behavioral Medicine* online.

kaae083_suppl_Supplementary_Tables_S2-S15

## Data Availability

De-identified data from this study are not available in a public archive. De-identified data from this study will be made available (as allowable according to institutional IRB standards) by emailing the corresponding author.
