# Supplementary material for: Effectiveness of behavior change techniques to address barriers to follow-up colonoscopy: results from an online survey and randomized factorial experiment
Source: Ann Behav Med. 2024 Dec 31;59(1):kaae083. doi: 10.1093/abm/kaae083 (PMC11761676; doi:10.1093/abm/kaae083)
Supplement: kaae083_suppl_Supplementary_Tables_S2-S15 [file kaae083_suppl_supplementary_tables_s2-s15.docx]

| **ESM Table 1.** TDF mapping exercise (reviewers 1 and 2) | | | | | | | | | | | | | | | |
| --- | --- | --- | --- | --- | --- | --- | --- | --- | --- | --- | --- | --- | --- | --- | --- |
| **Factors identified in Kerrison et al., 2021 & 2022** | **PAPM** | **Kn** | **Sk** | **SPRI** | **BaCa** | **Op** | **BaCo** | **Re** | **In** | **Go** | **MADP** | **ECR** | **SI** | **Em** | **BR** |
| **Sociocultural factors** | | | | | | | | | | | | | | | |
| **1.1. External influences** | | | | | | | | | | | | | | | |
| *1.1.1. Media coverage* | **1-6** | **1** | **0** | **0** | **0** | **0** | **0** | **2** | **0** | **0** | **0** | **0** | **0** | **0** | **0** |
| *1.1.2. Knowing someone with CRC* | **1-6** | **2** | **0** | **0** | **0** | **2** | **2** | **0** | **0** | **0** | **0** | **0** | **1** | **0** | **0** |
| *1.1.3. GP recommendation* | **1-6** | **0** | **0** | **1** | **0** | **0** | **0** | **2** | **0** | **0** | **0** | **0** | **2** | **0** | **0** |
| ***1.2. Individual, cultural and religious beliefs and attitudes*** | | | | | | | | | | | | | | | |
| 1*.2.1. Unable to have a male practitioner* | **3-4** | **0** | **0** | **2** | **0** | **0** | **0** | **0** | **2** | **0** | **0** | **0** | **2** | **0** | **0** |
| *1.2.2. Colonoscopy, colon and rectum ‘culturally taboo’ topics* | **1-4** | **0** | **0** | **1** | **0** | **0** | **0** | **0** | **0** | **0** | **0** | **2** | **1** | **0** | **0** |
| *1.2.3. Gender and engagement with healthcare* | **3-4** | **0** | **0** | **2** | **0** | **0** | **0** | **0** | **0** | **0** | **0** | **0** | **2** | **0** | **0** |
| *1.2.4. Fatalistic beliefs* | **3-4** | **1** | **0** | **0** | **0** | **2** | **2** | **0** | **0** | **0** | **0** | **0** | **0** | **1** | **0** |
| 1*.2.5. Unable to accept blood products* | **3-4** | **0** | **0** | **1** | **0** | **0** | **0** | **0** | **2** | **0** | **0** | **1** | **0** | **0** | **0** |
| *1.2.6. Attitudes towards free healthcare, regular health checks, healthcare professionals and healthcare provision in the UK* | **2-6** | **2** | **0** | **0** | **0** | **0** | **2** | **0** | **0** | **0** | **0** | **0** | **0** | **0** | **0** |
| *1.2.7. Lack of trust in Western Medicine* | **3-4** | **1** | **0** | **0** | **0** | **2** | **2** | **0** | **1** | **0** | **0** | **0** | **0** | **1** | **0** |
| *1.2.8. Valuing health* | **3-5, 3-6** | **0** | **0** | **1** | **0** | **0** | **2** | **0** | **1** | **2** | **0** | **0** | **0** | **0** | **0** |
| ***1.3. Past experiences and experiences of important others*** | | | | | | | | | | | | | | | |
| *1.3.1 Hearing other people’s experiences with colonoscopy* | **3-4, 3-5** | **2** | **0** | **0** | **0** | **2** | **2** | **2** | **0** | **0** | **0** | **0** | **2** | **0** | **0** |
| *1.3.2. Personal and family experiences with colonoscopy* | **3-4, 3-5** | **2** | **0** | **0** | **0** | **2** | **2** | **2** | **0** | **0** | **0** | **0** | **2** | **1** | **0** |
| ***1.4. Reliance on family and friends*** | | | | | | | | | | | | | | | |
| *1.4.1. Reliance on family and friends as unofficial interpreters* | **5-6** | **0** | **2** | **0** | **0** | **0** | **0** | **2** | **2** | **0** | **0** | **0** | **2** | **0** | **0** |
| *1.4.2. Reliance on family for travel & transport* | **5-6** | **0** | **2** | **0** | **0** | **0** | **0** | **2** | **2** | **0** | **0** | **0** | **2** | **0** | **0** |
| *1.4.3. Reliance on family for emotional support* | **5-6** | **0** | **0** | **0** | **0** | **0** | **1** | **2** | **2** | **0** | **0** | **0** | **2** | **1** | **0** |
| ***Practical factors*** | | | | | | | | | | | | | | | |
| ***2.1. Language barriers*** | | | | | | | | | | | | | | | |
| *2.1.1. Language barriers* | **1-6** | 0 | **2** | **0** | **0** | **0** | **0** | **0** | **2** | **0** | **0** | **0** | **0** | **0** | **0** |
| ***2.2. Competing priorities and accessibility issues*** | | | | | | | | | | | | | | | |
| *2.2.1. Transport / travel* | **5-6** | **0** | **2** | **0** | **0** | **0** | **0** | **0** | **2** | **0** | **0** | **2** | **0** | **0** | **0** |
| *2.2.2. Travelling / on holiday* | **5-6** | **0** | **0** | **0** | **0** | **0** | **0** | **0** | **0** | **2** | **0** | **2** | **0** | **0** | **0** |
| *2.2.3. Family, work and religious commitments* | **5-6** | **0** | **0** | **0** | **0** | **0** | **0** | **0** | **0** | **2** | **0** | **2** | **2** | **0** | **0** |
| *2.2.4. Lack of car parking* | **5-6** | **0** | **0** | **0** | **0** | **0** | **0** | **0** | **0** | **0** | **0** | **2** | **0** | **0** | **0** |
| *2.2.5. Indirect costs* | **5-6** | **0** | **0** | **0** | **0** | **0** | **1** | **0** | **0** | **0** | **0** | **2** | **0** | **0** | **0** |
| *2.2.6. Initial invitation not received* | **1-2** | **0** | **0** | **0** | **0** | **0** | **0** | **0** | **0** | **0** | **0** | **1** | **0** | **0** | **0** |

| **Factors identified in Kerrison et al., 2021 & 2022** | **PAPM** | **Kn** | **Sk** | **SPRI** | **BaCa** | **Op** | **BaCo** | **Re** | **In** | **Go** | **MADP** | **ECR** | **SI** | **Em** | **BR** |
| --- | --- | --- | --- | --- | --- | --- | --- | --- | --- | --- | --- | --- | --- | --- | --- |
| ***2.3. Unexpected events on the day of the appointment*** | | | | | | | | | | | | | | | |
| *2.3.1. Failed prep* | **5-6** | **0** | **2** | **0** | **0** | **0** | **0** | **0** | **2** | **0** | **0** | **1** | **0** | **0** | **0** |
| *2.3.2. Unwell* | **5-6** | **0** | **0** | **0** | **0** | **0** | **0** | **0** | **2** | **0** | **0** | **0** | **0** | **0** | **0** |
| *2.3.3. Personal emergency* | **5-6** | **0** | **0** | **0** | **0** | **0** | **0** | **0** | **2** | **0** | **0** | **1** | **0** | **0** | **0** |
| ***Psychological factors*** | | | | | | | | | | | | | | | |
| ***3.1. Concerns about the procedure*** | | | | | | | | | | | | | | | |
| 3.1.1. Concerns about doing the bowel preparation | **3-4, 3-5** | **2** | **2** | **0** | **2** | **0** | **2** | **0** | **0** | **0** | **0** | **0** | **0** | **0** | **0** |
| 3.1.2. Fear of pain and discomfort | **3-4, 3-5** | **2** | **0** | **0** | **0** | **1** | **2** | **0** | **0** | **0** | **0** | **0** | **0** | **0** | **0** |
| 3.1.3. Concerns about test invasiveness | **3-4, 3-5** | **2** | **0** | **0** | **1** | **0** | **2** | **0** | **0** | **0** | **0** | **0** | **0** | **0** | **0** |
| 3.1.4. Shame and embarrassment | **3-4, 3-5** | **1** | **0** | **0** | **0** | **0** | **1** | **0** | **1** | **0** | **0** | **0** | **0** | **0** | **0** |
| 3.1.5. Concerns about availability and necessity of sedation | **3-4, 3-5** | **2** | **0** | **0** | **1** | **0** | **1** | **0** | **0** | **0** | **0** | **0** | **0** | **0** | **0** |
| 3.1.6. Concerns about perforation and procedural risks | **3-4, 3-5** | **2** | **0** | **0** | **2** | **1** | **2** | **0** | **0** | **0** | **0** | **0** | **0** | **0** | **0** |
| 3.1.7. Concerns about practitioner performing the test | **3-4, 3-5** | **1** | **1** | **0** | **2** | **0** | **2** | **0** | **0** | **0** | **0** | **0** | **0** | **0** | **0** |
| ***3.2. Knowledge about CRC, screening and colonoscopy*** | | | | | | | | | | | | | | | |
| *3.2.1. Lack of understanding that bowel cancer can be asymptomatic and the test is looking for invisible traces of blood* | **1-5** | **2** | **0** | **0** | **0** | **0** | **2** | **0** | **0** | **0** | **0** | **0** | **0** | **0** | **0** |
| *3.2.2. Lack of awareness and understanding of colonoscopy procedure* | **1-5** | **2** | **0** | **0** | **0** | **0** | **2** | **0** | **0** | **0** | **0** | **0** | **0** | **0** | **0** |
| ***3.3. Emotional responses during the assessment*** | | | | | | | | | | | | | | | |
| *3.3.1. Anxiety* | **1-5** | **0** | **0** | **0** | **1** | **0** | **0** | **0** | **0** | **0** | **0** | **0** | **0** | **2** | **2** |
| *3.3.2. Denial* | **2-4** | **0** | **0** | **0** | **0** | **0** | **0** | **0** | **0** | **0** | **0** | **0** | **0** | **2** | **2** |
| *3.3.3. Avoidance* | **2-4** | **0** | **0** | **0** | **1** | **0** | **0** | **0** | **0** | **0** | **0** | **0** | **0** | **2** | **2** |
| *3.3.4. Shock* | **2-4** | **0** | **0** | **0** | **0** | **0** | **0** | **0** | **0** | **0** | **0** | **0** | **0** | **2** | **2** |
| ***3.4. Cognitive abilities and ability to make an informed decision*** | | | | | | | | | | | | | | | |
| *3.4.1. Lack of capacity* | **5-6** | **1** | **2** | **0** | **0** | **0** | **0** | **0** | **0** | **0** | **2** | **0** | **0** | **0** | **0** |
| *3.4.2. Low health literacy* | **1-5** | **2** | **2** | **0** | **1** | **0** | **2** | **0** | **0** | **0** | **2** | **0** | **0** | **0** | **0** |
| *3.4.3. Memory issues* | **1-6** | **1** | **0** | **0** | **0** | **0** | **0** | **0** | **0** | **0** | **2** | **0** | **0** | **0** | **0** |
| ***3.5. Perceived CRC risk and perceived benefits of colonoscopy*** | | | | | | | | | | | | | | | |
| 3.5.1. Peace of mind | **3-6** | **1** | **0** | **0** | **0** | **2** | **2** | **1** | **2** | **2** | **0** | **0** | **0** | **0** | **0** |
| 3.5.2. Having CRC symptoms | **2-6** | **2** | **0** | **0** | **0** | **0** | **0** | **0** | **0** | **0** | **0** | **0** | **0** | **0** | **0** |
| 3.5.3. Personal or family history of CRC | **2-6** | **2** | **0** | **0** | **0** | **2** | **2** | **2** | **2** | **2** | **0** | **2** | **1** | **1** | **0** |
| **Factors identified in Kerrison et al., 2021 & 2022** | **PAPM** | **Kn** | **Sk** | **SPRI** | **BaCa** | **Op** | **BaCo** | **Re** | **In** | **Go** | **MADP** | **ECR** | **SI** | **Em** | **BR** |
| *3.5.4.1. Fear of cancer*  *3.5.4.2. Belief that cancer is a treatable disease* | **2-5** | **2** | **0** | **0** | **0** | **2** | **2** | **0** | **2** | **2** | **0** | **0** | **0** | **2** | **0** |
| *3.5.5. Perception of colonoscopy as life-saving* | **2-6** | **2** | **0** | **0** | **0** | **2** | **2** | **1** | **2** | **2** | **0** | **0** | **0** | **0** | **0** |
| *3.5.6. Perceived importance of screening* | **3-5, 3-6** | **1** | **0** | **0** | **0** | **0** | **2** | **0** | **2** | **0** | **0** | **1** | **0** | **0** | **0** |

| **Factors identified in Kerrison et al., 2021 & 2022** | **PAPM** | **Kn** | **Sk** | **SPRI** | **BaCa** | **Op** | **BaCo** | **Re** | **In** | **Go** | **MADP** | **ECR** | **SI** | **Em** | **BR** |
| --- | --- | --- | --- | --- | --- | --- | --- | --- | --- | --- | --- | --- | --- | --- | --- |
| ***3.9. Locus of control*** | | | | | | | | | | | | | | | |
| *3.9.1. Free will / personal choice in medical decision making* | **3-5, 3-6** | **0** | **0** | **0** | **2** | **0** | **0** | **0** | **0** | **0** | **2** | **0** | **0** | **0** | **0** |
| *3.9.2. Obtaining detailed information facilitates participation* | **2-6** | **2** | **0** | **0** | **2** | **0** | **2** | **0** | **0** | **0** | **1** | **2** | **0** | **0** | **0** |
| *3.9.3. Reliance on medical professional / authority* | **3-5, 3-6** | **0** | **0** | **1** | **1** | **0** | **0** | **1** | **0** | **0** | **0** | **0** | **2** | **0** | **0** |
| *3.9.4. Shared decision making and family influenced participation* | **3-5, 3-6** | **1** | **0** | **0** | **2** | **0** | **1** | **1** | **2** | **0** | **2** | **1** | **2** | **0** | **0** |
| *3.9.5. Religious faith facilitates participation* | **3-5, 3-6** | **0** | **0** | **0** | **2** | **0** | **2** | **1** | **2** | **2** | **0** | **2** | **2** | **0** | **0** |
| *3.9.6. The role of God in determining the future* | **3-5, 3-6** | **1** | **0** | **0** | **2** | **0** | **2** | **1** | **1** | **0** | **2** | **0** | **1** | **0** | **0** |
| ***Health-related factors*** | | | | | | | | | | | | | | | |
| ***4.1. Clinically ineligible or inappropriate*** | | | | | | | | | | | | | | | |
| *4.1.1. Clinically ineligible or inappropriate* | **5-6** | **0** | **0** | **0** | **0** | **0** | **0** | **0** | **0** | **0** | **0** | **0** | **0** | **0** | **0** |
| ***4.2. Factors affecting patient willingness to have the test*** | | | | | | | | | | | | | | | |
| *4.2.1. Recent Colonoscopy* | **3-5, 3-6** | **0** | **0** | **0** | **2** | **2** | **2** | **0** | **2** | **0** | **0** | **2** | **0** | **0** | **0** |
| *4.2.2. Existing health conditions interfering with ability to complete procedure or bowel prep* | **3-5** | **0** | **2** | **0** | **2** | **0** | **1** | **0** | **0** | **0** | **1** | **0** | **0** | **0** | **0** |
| *4.2.3. Previous personal experiences with colonoscopy and other medical investigations* | **3-5, 3-6** | **2** | **0** | **0** | **2** | **0** | **2** | **0** | **2** | **0** | **0** | **2** | **0** | **0** | **0** |
| *4.2.4. Existing health conditions as a competing priority* | **3-5** | **0** | **0** | **0** | **2** | **0** | **1** | **0** | **0** | **0** | **0** | **2** | **0** | **0** | **0** |
| 0 = Barrrier / facilitator assigned to respective TDF domain by neither reviewer  1 = Barrier / facilitator assigned to respective TDF domain by one reviewer  2 = Barrier / facilitator assigned to respective TDF domain by both reviewers | | | | | | | | | | | | | | | |

| **ESM 2.** Evidence matrix for the effectiveness of BCTs to target individual TDF domains | | | | | | | | | | | | | | |
| --- | --- | --- | --- | --- | --- | --- | --- | --- | --- | --- | --- | --- | --- | --- |
| **Factors identified in Kerrison et al., 2021 & 2022** | **Kn** | **Sk** | **SPRI** | **BaCa** | **Op** | **BaCo** | **Re** | **In** | **Go** | **MADP** | **ECR** | **SI** | **Em** | **BR** |
| 1.1. Goal setting (behaviour) |  |  |  |  |  |  |  |  |  |  |  |  |  |  |
| 1.2. Problem solving |  |  |  |  |  |  |  |  |  |  |  |  |  |  |
| 1.3. Goal setting (outcome) |  |  |  |  |  |  |  |  |  |  |  |  |  |  |
| 1.4. Action planning |  |  |  |  |  |  |  |  |  |  |  |  |  |  |
| 1.5. Review behaviour goal(s) |  |  |  |  |  |  |  |  |  |  |  |  |  |  |
| 1.6. Discrepancy between current behaviour and goal |  |  |  |  |  |  |  |  |  |  |  |  |  |  |
| 1.7. Review outcome goal(s) |  |  |  |  |  |  |  |  |  |  |  |  |  |  |
| 1.8. Behavioural contract |  |  |  |  |  |  |  |  |  |  |  |  |  |  |
| 1.9. Commitment |  |  |  |  |  |  |  |  |  |  |  |  |  |  |
| 2.1. Monitoring of behaviour by others without feedback |  |  |  |  |  |  |  |  |  |  |  |  |  |  |
| 2.2. Feedback on behaviour |  |  |  |  |  |  |  |  |  |  |  |  |  |  |
| 2.3. Self-monitoring of behaviour |  |  |  |  |  |  |  |  |  |  |  |  |  |  |
| 2.4. Self-monitoring of outcomes of behaviour |  |  |  |  |  |  |  |  |  |  |  |  |  |  |
| 2.5. Monitoring of outcome(s) of behaviour without feedback |  |  |  |  |  |  |  |  |  |  |  |  |  |  |
| 2.6. Biofeedback |  |  |  |  |  |  |  |  |  |  |  |  |  |  |
| 2.7. Feedback on outcome(s) of behaviour |  |  |  |  |  |  |  |  |  |  |  |  |  |  |
| 3.1. Social support (unspecified) |  |  |  |  |  |  |  |  |  |  |  |  |  |  |
| 3.2. Social support (practical) |  |  |  |  |  |  |  |  |  |  |  |  |  |  |
| 3.3. Social support (emotional) |  |  |  |  |  |  |  |  |  |  |  |  |  |  |
| 4.1. Instruction on how to perform behaviour |  |  |  |  |  |  |  |  |  |  |  |  |  |  |
| 4.2. Information about antecedents |  |  |  |  |  |  |  |  |  |  |  |  |  |  |
| 4.3. Re-attribution |  |  |  |  |  |  |  |  |  |  |  |  |  |  |
| 5.1. Information about health consequences |  |  |  |  |  |  |  |  |  |  |  |  |  |  |
| 5.2. Salience of consequences |  |  |  |  |  |  |  |  |  |  |  |  |  |  |
| 5.3. Information about social and environmental consequences |  |  |  |  |  |  |  |  |  |  |  |  |  |  |
| 5.4. Monitoring of emotional consequences |  |  |  |  |  |  |  |  |  |  |  |  |  |  |
| 5.5. Anticipated regret |  |  |  |  |  |  |  |  |  |  |  |  |  |  |
| 5.6. Information about emotional consequences |  |  |  |  |  |  |  |  |  |  |  |  |  |  |
| 6.1. Demonstration of the behaviour |  |  |  |  |  |  |  |  |  |  |  |  |  |  |
| 6.2. Social comparison |  |  |  |  |  |  |  |  |  |  |  |  |  |  |
| 6.3. Information about others’ approval |  |  |  |  |  |  |  |  |  |  |  |  |  |  |
| 7.1. Prompts/cues |  |  |  |  |  |  |  |  |  |  |  |  |  |  |
| 7.5. Remove aversive stimulus |  |  |  |  |  |  |  |  |  |  |  |  |  |  |
| 7.7. Exposure |  |  |  |  |  |  |  |  |  |  |  |  |  |  |
| 7.8. Associative learning |  |  |  |  |  |  |  |  |  |  |  |  |  |  |
| 8.1. Behavioural practice/rehearsal |  |  |  |  |  |  |  |  |  |  |  |  |  |  |
| 8.2. Behaviour substitution |  |  |  |  |  |  |  |  |  |  |  |  |  |  |
| 8.3. Habit formation |  |  |  |  |  |  |  |  |  |  |  |  |  |  |
| 8.4. Habit reversal |  |  |  |  |  |  |  |  |  |  |  |  |  |  |
| 8.6. Generalisation of target behaviour |  |  |  |  |  |  |  |  |  |  |  |  |  |  |
| 8.7. Graded tasks |  |  |  |  |  |  |  |  |  |  |  |  |  |  |
| 9.1. Credible source |  |  |  |  |  |  |  |  |  |  |  |  |  |  |
| 9.2. Pros and cons |  |  |  |  |  |  |  |  |  |  |  |  |  |  |
| 9.3. Comparative imagining of future outcomes |  |  |  |  |  |  |  |  |  |  |  |  |  |  |
| 10.1. Material incentive (behaviour) |  |  |  |  |  |  |  |  |  |  |  |  |  |  |
| 10.2. Material reward (behaviour) |  |  |  |  |  |  |  |  |  |  |  |  |  |  |
| 10.3. Non-specific reward |  |  |  |  |  |  |  |  |  |  |  |  |  |  |
| 10.4. Social reward |  |  |  |  |  |  |  |  |  |  |  |  |  |  |
| 10.6. Non-specific incentive |  |  |  |  |  |  |  |  |  |  |  |  |  |  |
| 10.7. Self-incentive |  |  |  |  |  |  |  |  |  |  |  |  |  |  |
| 10.8. Incentive (outcome) |  |  |  |  |  |  |  |  |  |  |  |  |  |  |
| 10.9. Self-reward |  |  |  |  |  |  |  |  |  |  |  |  |  |  |
| 10.10. Reward (outcome) |  |  |  |  |  |  |  |  |  |  |  |  |  |  |
| 11.1. Pharmacological support |  |  |  |  |  |  |  |  |  |  |  |  |  |  |
| 11.2. Reduce negative emotions |  |  |  |  |  |  |  |  |  |  |  |  |  |  |
| 11.3. Conserving mental resources |  |  |  |  |  |  |  |  |  |  |  |  |  |  |
| 11.4. Paradoxical instructions |  |  |  |  |  |  |  |  |  |  |  |  |  |  |
| 12.1. Restructuring the physical environment |  |  |  |  |  |  |  |  |  |  |  |  |  |  |
| 12.2. Restructuring the social environment |  |  |  |  |  |  |  |  |  |  |  |  |  |  |
| 12.3. Avoidance/reducing exposure to cues for the behaviour |  |  |  |  |  |  |  |  |  |  |  |  |  |  |
| 12.5. Adding objects to the environment |  |  |  |  |  |  |  |  |  |  |  |  |  |  |
| 12.6. Body changes |  |  |  |  |  |  |  |  |  |  |  |  |  |  |
| 13.1. Identification of self as role model |  |  |  |  |  |  |  |  |  |  |  |  |  |  |
| 13.2. Framing/reframing |  |  |  |  |  |  |  |  |  |  |  |  |  |  |
| 13.3. Incompatible beliefs |  |  |  |  |  |  |  |  |  |  |  |  |  |  |
| 13.4. Valued self-identify |  |  |  |  |  |  |  |  |  |  |  |  |  |  |
| 13.5. Identity associated with changed behaviour |  |  |  |  |  |  |  |  |  |  |  |  |  |  |
| 14.2. Punishment |  |  |  |  |  |  |  |  |  |  |  |  |  |  |
| 15.1. Verbal persuasion about capability |  |  |  |  |  |  |  |  |  |  |  |  |  |  |
| 15.2. Mental rehearsal of successful performance |  |  |  |  |  |  |  |  |  |  |  |  |  |  |
| 15.3. Focus on past success |  |  |  |  |  |  |  |  |  |  |  |  |  |  |
| 15.4. Self-talk |  |  |  |  |  |  |  |  |  |  |  |  |  |  |
| 16.2. Imaginary reward |  |  |  |  |  |  |  |  |  |  |  |  |  |  |
| 16.3. Vicarious consequences |  |  |  |  |  |  |  |  |  |  |  |  |  |  |
| Cells highlighted in green indicate strong evidence to support the use of the corresponding BCT to target the respective domain, while those in amber indicate those with mixed evidence, and those left blank no evidence. | | | | | | | | | | | | | | |

| **ESM Table 3a.** Excluded BCTs (No evidence) |
| --- |
| 1. 2.3. Self-monitoring of behaviour |
| 1. 2.7. Feedback on outcome(s) of behaviour |
| 1. 3.3. Social support (emotional) |
| 1. 4.3. Re-attribution |
| 1. 5.4. Monitoring of emotional consequences |
| 1. 7.7. Exposure |
| 1. 10.7. Self-incentive |
| 1. 10.9. Self-reward |
| 1. 11.1. Pharmacological support |
| 1. 11.4. Paradoxical instructions |
| 1. 13.1. Identification of self as role model |
| 1. 13.3. Incompatible beliefs |
| 1. 15.2. Mental rehearsal of successful performance |
| 1. 16.2. Imaginary reward |
| 1. 16.3. Vicarious consequences |

| **ESM Table 3b.** Excluded BCTs (Inconclusive evidence) |
| --- |
| 1. 1.4. Action planning |
| 1. 1.8. Behavioural contract |
| 1. 1.9. Commitment |
| 1. 2.1. Monitoring of behaviour by others without feedback |
| 1. 2.2. Feedback on behaviour |
| 1. 2.5. Monitoring of outcome(s) of behaviour without feedback |
| 1. 7.8. Associative learning |
| 1. 8.3. Habit formation |
| 1. 8.4. Habit reversal |
| 1. 8.6. Generalisation of target behaviour |
| 1. 9.1. Credible source |
| 1. 12.6. Body changes |
| 1. 13.2. Framing/reframing |
| 1. 13.4. Valued self-identify |
| 1. 13.5. Identity associated with changed behaviour |

| **ESM Table 3c.** Excluded BCTs (not relevant to target behaviour) |
| --- |
| 1. 1.1. Goal setting (behaviour) |
| 1. 1.5. Review behaviour goal(s) |
| 1. 1.6. Discrepancy between current behaviour and goal |
| 1. 1.7. Review outcome goal(s) |
| 1. 2.4. Self-monitoring of outcomes of behaviour |
| 1. 2.6. Biofeedback |
| 1. 4.2. Information about antecedents |
| 1. 5.3. Information about social and environmental consequences |
| 1. 7.1. Prompts/cues |
| 1. 7.5. Remove aversive stimulus |
| 1. 8.1. Behavioural practice/rehearsal |
| 1. 8.2. Behaviour substitution |
| 1. 8.7. Graded tasks |
| 1. 10.1. Material incentive (behaviour) |
| 1. 10.2. Material reward (behaviour) |
| 1. 10.3. Non-specific reward |
| 1. 10.4. Social reward |
| 1. 10.6. Non-specific incentive |
| 1. 10.8. Incentive (outcome) |
| 1. 10.10. Reward (outcome) |
| 1. 11.2. Reduce negative emotions |
| 1. 11.3. Conserving mental resources |
| 1. 12.1. Restructuring the physical environment |
| 1. 12.2. Restructuring the social environment |
| 1. 12.3. Avoidance/reducing exposure to cues for the behaviour |
| 1. 12.5. Adding objects to the environment |
| 1. 14.2. Punishment |
| 1. 15.3. Focus on past success |

| **ESM Table 3d.** Excluded BCTs (Incongruent with the ethos of informed decision making for target behaviour) |
| --- |
| 5.5. Anticipated regret |
| 5.6. Information about emotional consequences |
| 9.3. Comparative imagining of future outcomes |

| **ESM Table 3e.** Excluded barriers and facilitators (excluded on the basis that they had not been assigned a domain that could be targeted by any one of the selected BCTs) |
| --- |
| *1.2.6. Attitudes towards free healthcare, regular health checks, healthcare professionals and healthcare provision in the UK* |
| *2.3.1. Failed prep* |
| *2.3.2. Unwell* |
| *2.3.3. Personal emergency* |
| *3.4.3. Memory issues* |
| *3.4.1. Lack of capacity* |
| *4.1.1. Clinically ineligible or inappropriate* |

| **ESM Table 3f.** Excluded barriers and facilitators (not addressable via appointment letter) |
| --- |
| *1.1.1. Media coverage* |
| *1.1.2. Knowing someone with CRC* |
| 3.5.2. Having CRC symptoms |
| *2.2.6. Initial invitation not received* |
| *2.3.1. Failed prep* |
| *2.3.2. Unwell* |
| *2.3.3. Personal emergency* |
| *3.4.1. Lack of capacity* |
| *4.1.1. Clinically ineligible or inappropriate* |

| **ESM Table 3g.** Excluded barriers and facilitators (‘duplicates’ of other barriers or facilitators) |
| --- |
| *3.1.7. Concerns about practitioner performing the test* |

| **ESM Table 3h.** Excluded barriers and facilitators (deemed ‘facilitators’, and not targetable by BCTs) |
| --- |
| *1.1.3. GP recommendation* |
| *1.2.8. Valuing health* |
| 3.5.1. Peace of mind |
| 3.5.3. Personal or family history of CRC |
| *3.5.5. Perception of colonoscopy as life-saving* |
| *3.9.5. Religious faith facilitates participation* |
| *3.9.1. Free will / personal choice in medical decision making* |
| *3.9.2. Obtaining detailed information facilitates participation* |

| **ESM Table 3i.** Excluded barriers (no conclusive BCTs to address these barriers) |
| --- |
| 1*.2.1. Unable to have a male practitioner* |
| *1.3.2. Personal and family experiences with colonoscopy* |
| *2.2.2. Travelling / on holiday* |
| *2.2.3. Family, work and religious commitments* |
| *3.1.5. Concerns about availability and necessity of sedation* |
| *3.1.6. Concerns about perforation and procedural risks* |
| *4.2.1. Recent Colonoscopy* |
| *4.2.3. Previous personal experiences with colonoscopy and other medical investigations* |
| *4.2.4. Existing health conditions as a competing priority* |

| **ESM Table 4.** Selected BCTs for further consideration (and the level of evidence to support their use) | | | | | | | | | | | | | | | |
| --- | --- | --- | --- | --- | --- | --- | --- | --- | --- | --- | --- | --- | --- | --- | --- |
| **Factors identified in Kerrison et al., 2021 & 2022** | **Relevant** | **Kn** | **Sk** | **SPRI** | **BaCa** | **Op** | **BaCo** | **Re** | **In** | **Go** | **MADP** | **ECR** | **SI** | **Em** | **BR** |
| 1.2. Problem solving | Yes |  |  |  | **X** |  |  |  |  |  |  |  |  |  | **X** |
| 3.1. Social support (unspecified) | Yes |  |  |  |  |  |  |  |  |  |  |  | **X** |  |  |
| 3.2. Social support (practical) | Yes |  |  |  |  |  |  |  |  |  |  | **X** | **X** |  |  |
| 4.1. Instruction on how to perform behaviour | Yes | **X** | **X** |  | **X** |  |  |  |  |  |  |  |  |  |  |
| 5.1. Information about health consequences | Yes | **X** |  |  |  |  | **X** |  | **X** |  |  |  |  |  |  |
| 5.2. Salience of consequences | Yes |  |  |  |  |  | **X** |  |  |  |  |  |  |  |  |
| 6.1. Demonstration of the behaviour | Yes |  |  |  | **X** |  |  |  |  |  |  |  |  |  |  |
| 6.2. Social comparison | Yes |  |  |  |  |  |  |  |  |  |  |  | **X** |  |  |
| 6.3. Information about others’ approval | Yes |  |  |  |  |  |  |  |  |  |  |  | **X** |  |  |
| 9.2. Pros and cons | Yes |  |  |  |  |  | **X** |  |  |  |  |  |  |  |  |
| 11.2. Reduce negative emotions | Yes |  |  |  |  |  |  |  |  |  |  |  |  | **X** |  |
| 15.1. Verbal persuasion about capability | Yes |  |  |  | **X** |  |  |  |  |  |  |  |  |  |  |
| 15.4. Self-talk | Yes |  |  |  | **X** |  |  |  |  |  |  |  |  |  |  |
| Green = conclusive evidence  Orange = Inconclusive evidence  White = No evidence | | | | | | | | | | | | | | | |

| **ESM Table 5.** Allocation of barriers and facilitators to a single TDF domain and construct. | | | | | | | | | | |
| --- | --- | --- | --- | --- | --- | --- | --- | --- | --- | --- |
| **Factors identified in Kerrison et al., 2021 & 2022** | **PAPM** | **Kn** | **BaCa** | **BaCo** | **In** | **ECR** | **Sk** | **SI** | **BR** | **Em** |
| **1.1. Knowledge (Knowledge of condition / scientific rationale)** | | | | | | | | | | |
| *3.2.1. Lack of understanding that bowel cancer can be asymptomatic and the test is looking for invisible traces of blood* | **1-5** | **2** | **0** | **2** | **0** | **0** | **0** | **0** | **0** | **0** |
| 3.5.3. Personal or family history of CRC | **2-6** | **2** | **0** | **2** | **2** | **2** | **0** | **1** | **0** | **0** |
| *3.5.4.2. Belief that cancer is a treatable disease* | **2-5** | **2** | **0** | **2** | **2** | **0** | **0** | **0** | **0** | **0** |
| **1.2. Knowledge (Procedural knowledge)** | | | | | | | | | | |
| *1.3.1 Hearing other people’s experiences with colonoscopy* | **3-4, 3-5** | **2** | **0** | **2** | **0** | **0** | **0** | **2** | **0** | **0** |
| *1.3.2. Personal and family experiences with colonoscopy* | **3-4, 3-5** | **2** | **0** | **2** | **0** | **0** | **0** | **2** | **0** | **0** |
| *3.9.2. Obtaining detailed information facilitates participation* | **2-6** | **2** | **2** | **2** | **0** | **2** | **0** | **0** | **0** | **0** |
| *3.2.2. Lack of awareness and understanding of colonoscopy procedure* | **1-5** | **2** | **0** | **2** | **0** | **0** | **0** | **0** | **0** | **0** |
| *4.2.3. Previous personal experiences with colonoscopy and other medical investigations* | **3-5, 3-6** | **2** | **2** | **2** | **2** | **2** | **0** | **0** | **0** | **0** |
| **1.3. Knowledge (Knowledge of task and environment)** | | | | | | | | | | |
| *-* | **-** | **-** | **-** | **-** | **-** | **-** | **-** | **-** | **-** | **-** |
| **2.1. Beliefs about capabilities (Self-confidence)** | | | | | | | | | | |
| *-* | **-** | **-** | **-** | **-** | **-** | **-** | **-** | **-** | **-** | **-** |
| **2.2. Beliefs about capabilities (Perceived competence)** | | | | | | | | | | |
| *-* | **-** | **-** | **-** | **-** | **-** | **-** | **-** | **-** | **-** | **-** |
| **2.3. Beliefs about capabilities (Self-efficacy)** Perceived behavioural control, Beliefs, Self-esteem, Empowerment and Professional confidence | | | | | | | | | | |
| *4.2.2. Existing health conditions interfering with ability to complete procedure or bowel prep* | **3-5** | **0** | **2** | **1** | **0** | **0** | **2** | **0** | **0** | **0** |
| *4.2.1. Recent Colonoscopy* | **3-5, 3-6** | **0** | **2** | **2** | **2** | **2** | **0** | **0** | **0** |  |
| **2.4. Beliefs about capabilities (Perceived behavioural control)** | | | | | | | | | | |
| *3.9.1. Free will / personal choice in medical decision making* | **3-5, 3-6** | **0** | **2** | **0** | **0** | **0** | **0** | **0** | **0** | **0** |
| **2.5. Beliefs about capabilities (Beliefs)** | | | | | | | | | | |
| - | **-** | **-** | **-** | **-** | **-** | **-** | **-** | **-** | **-** | **-** |
| **2.6. Beliefs about capabilities (Self-esteem)** | | | | | | | | | | |
| - | **-** | **-** | **-** | **-** | **-** | **-** | - | **-** | **-** | **-** |
| **2.7. Beliefs about capabilities (Empowerment)** | | | | | | | | | | |
| - | **-** | **-** | **-** | **-** | **-** | **-** | **-** | **-** | **-** | **-** |
| **2.8. Beliefs about capabilities (Professional confidence)** | | | | | | | | | | |
| - | **-** | **-** | **-** | **-** | **-** | **-** | **-** | **-** | **-** | **-** |
| **3.1. Beliefs about consequences (Beliefs)** | | | | | | | | | | |
| *1.2.4. Fatalistic beliefs* | **3-4** | **1** | **0** | **2** | **0** | **0** | **0** | **0** | **0** | **0** |
| *1.2.7. Lack of trust in Western Medicine* | **3-4** | **1** | **0** | **2** | **1** | **0** | **0** | **0** | **0** | **0** |
| *1.2.8. Valuing health* | **3-5, 3-6** | **0** | **0** | **2** | **1** | **0** | **0** | **0** | **0** | **0** |
| *3.5.6. Perceived importance of screening* | **3-5, 3-6** | **1** | **0** | **2** | **2** | **1** | **0** | **0** | **0** | **0** |
| *3.9.5. Religious faith facilitates participation* | **3-5, 3-6** | **0** | **2** | **2** | **2** | **2** | **0** | **2** | **0** | **0** |
| *3.9.6. The role of God in determining the future* | **3-5, 3-6** | **1** | **2** | **2** | **1** | **0** | **0** | **1** | **0** | **0** |
| **3.2. Beliefs about consequences (Outcome expectancies)** | | | | | | | | | | |
| 3.5.1. Peace of mind | **3-6** | **1** | **0** | **2** | **2** | **0** | **0** | **0** | **0** | **0** |
| *3.5.5. Perception of colonoscopy as life-saving* | **2-6** | **2** | **0** | **2** | **2** | **0** | **0** | **0** | **0** | **0** |
| **3.3. Beliefs about consequences (Characteristics of outcome expectancies)** | | | | | | | | | | |
| - | **-** | **-** | **-** | **-** | **-** | **-** | **-** | **-** | **-** | **-** |
| **3.4. Beliefs about consequences (Anticipated regret)** | | | | | | | | | | |
| - | **-** | **-** | **-** | **-** | **-** | **-** | **-** | **-** | **-** | **-** |
| **3.5. Beliefs about consequences (Consequents)** | | | | | | | | | | |
| - | **-** | **-** | **-** | **-** | **-** | **-** | **-** | **-** | **-** | **-** |
| **4.1. Intentions (Stability of intentions)** | | | | | | | | | | |
| - | **-** | **-** | **-** | **-** | **-** | **-** | **-** | **-** | **-** | **-** |
| **4.2. Intentions (Stages of change mode)** | | | | | | | | | | |
| - | **-** | **-** | **-** | **-** | **-** | **-** | **-** | **-** | **-** | **-** |
| **4.3. Intentions (Transtheoretical model)** | | | | | | | | | | |
| - | **-** | **-** | **-** | **-** | **-** | **-** | **-** | **-** | **-** | **-** |
| **4.4. Intentions (Stages of change)** | | | | | | | | | | |
| - | **-** | **-** | **-** | **-** | **-** | **-** | **-** | **-** | **-** | **-** |
| **5.1. Environmental Context and Resources (Environmental stressors)** | | | | | | | | | | |
| - | **-** | **-** | **-** | **-** | **-** | **-** | **-** | **-** | **-** | **-** |
| **5.2. Environmental Context and Resources (Resources / material resources)** | | | | | | | | | | |
| *2.1.1. Language barriers* | **1-6** | **0** | **0** | **0** | **2** | **0** | **2** | **0** | **0** | **0** |
| **5.3. Environmental Context and Resources (Organisational culture / climate)** | | | | | | | | | | |
| - | **-** | **-** | **-** | **-** | **-** | **-** | **-** | **-** | **-** | **-** |
| **5.4. Environmental Context and Resources (Salient events / critical incidents)** | | | | | | | | | | |
| - | **-** | **-** | **-** | **-** | **-** | **-** | **-** | **-** | **-** | **-** |
| **5.5. Environmental Context and Resources (Person × environment interaction)** | | | | | | | | | | |
| *4.2.4. Existing health conditions as a competing priority* | **3-5** | **0** | **2** | **1** | **0** | **2** | **0** | **0** | **0** | **0** |
| **5.6. Environmental Context and Resources (Barriers and facilitators)** | | | | | | | | | | |
| *2.2.1. Transport / travel* | **5-6** | **0** | **0** | **0** | **2** | **2** | **2** | **0** | **0** | **0** |
| *2.2.2. Travelling / on holiday* | **5-6** | **0** | **0** | **0** | **0** | **2** | **0** | **0** | **0** | **0** |
| *2.2.3. Family, work and religious commitments* | **5-6** | **0** | **0** | **0** | **0** | **2** | **0** | **2** | **0** | **0** |
| *2.2.4. Lack of car parking* | **5-6** | **0** | **0** | **0** | **0** | **2** | **0** | **0** | **0** | **0** |
| *2.2.5. Indirect costs* | **5-6** | **0** | **0** | **1** | **0** | **2** | **0** | **0** | **0** | **0** |
| **6.1. Skills (Skills development)** | | | | | | | | | | |
| *-* | **-** | **-** | **-** | **-** | **-** | **-** | **-** | **-** | **-** | **-** |
| **6.2. Skills (Competence)** | | | | | | | | | | |
| *-* | **-** | **-** | **-** | **-** | **-** | **-** | **-** | **-** | **-** | **-** |
| **6.3. Skills (Ability)** | | | | | | | | | | |
| *3.4.2. Low health literacy* | **1-5** | **2** | **1** | **2** | **0** | **0** | **2** | **0** | **0** |  |
| **6.4. Skills (Interpersonal skills)** | | | | | | | | | | |
| *-* | **-** | **-** | **-** | **-** | **-** | **-** | **-** | **-** | **-** | **-** |
| **6.5. Skills (Practice)** | | | | | | | | | | |
| *-* | **-** | **-** | **-** | **-** | **-** | **-** | **-** | **-** | **-** | **-** |
| **6.6. Skills (Skill assessment)** | | | | | | | | | | |
| *-* | **-** | **-** | **-** | **-** | **-** | **-** | **-** | **-** | **-** | **-** |
| **7.1. Social Influences (Social pressure)** | | | | | | | | | | |
| *-* | **-** | - | **-** | **-** | **-** | **-** | **-** | **-** | **-** | **-** |
| **7.2. Social Influences (Social norms)** | | | | | | | | | | |
| 1*.2.1. Unable to have a male practitioner* | **3-4** | **0** | **0** | **0** | **2** | **0** | **0** | **2** | **0** | **0** |
| *1.2.2. Colonoscopy, colon and rectum ‘culturally taboo’ topics* | **1-4** | **0** | **0** | **0** | **0** | **2** | **0** | **1** | **0** | **0** |
| 1*.2.5. Unable to accept blood products* | **3-4** | **0** | **0** | **0** | **2** | **1** | **0** | **0** | **0** | **0** |
| **7.3. Social Influences (Group conformity)** | | | | | | | | | | |
| *-* | **-** | - | **-** | **-** | **-** | **-** | **-** | **-** | **-** | **-** |
| **7.4. Social Influences (Social comparisons)** | | | | | | | | | | |
| *-* | **-** | - | **-** | **-** | **-** | **-** | **-** | **-** | **-** | **-** |
| **7.5. Social Influences (Group norms**) | | | | | | | | | | |
| *1.2.3. Gender and engagement with healthcare* | **3-4** | **0** | **0** | **0** | **0** | **0** | **0** | **2** | **0** | **0** |
| **7.6. Social Influences (Social support)** | | | | | | | | | | |
| *1.4.1. Reliance on family and friends as unofficial interpreters* | **5-6** | **0** | **0** | **0** | **2** | **0** | **2** | **2** | **0** | **0** |
| *1.4.2. Reliance on family for travel & transport* | **5-6** | **0** | **0** | **0** | **2** | **0** | **2** | **2** | **0** | **0** |
| *1.4.3. Reliance on family for emotional support* | **5-6** | **0** | **0** | **1** | **2** | **0** | **0** | **2** | **0** | **0** |
| *3.9.4. Shared decision making and family influenced participation* | **3-5, 3-6** | **1** | **2** | **1** | **2** | **1** | **0** | **2** | **0** | **0** |
| **7.7. Social Influences (Power)** | | | | | | | | | | |
| *1.1.3. GP recommendation* | **1-6** | **0** | **0** | **0** | **0** | **0** | **0** | **2** | **0** | **0** |
| *3.9.3. Reliance on medical professional / authority* | **3-5, 3-6** | **0** | **1** | **0** | **0** | **0** | **0** | **2** | **0** | **0** |
| **7.8. Social Influences (Inter group conflict)** | | | | | | | | | | |
| *-* | **-** | - | **-** | **-** | **-** | **-** | **-** | **-** | **-** | **-** |
| **7.9. Social Influences (Alienation)** | | | | | | | | | | |
| *-* | **-** | - | **-** | **-** | **-** | **-** | **-** | **-** | **-** | **-** |
| **7.10. Social Influences (Group identity)** | | | | | | | | | | |
| *-* | **-** | - | **-** | **-** | **-** | **-** | **-** | **-** | **-** | **-** |
| **7.11. Social Influences (Modelling)** | | | | | | | | | | |
| *-* | **-** | - | **-** | **-** | **-** | **-** | **-** | **-** | **-** | **-** |
| **8.1. Emotion (Fear)** | | | | | | | | | | |
| *3.1.2. Fear of pain and discomfort* | **3-4, 3-5** | **2** | **0** | **2** | **0** | **0** | **0** | **0** | **0** | **2** |
| *3.5.4.1. Fear of cancer* | **2-5** | **2** | **0** | **2** | **2** | **0** | **0** | **0** | **0** | **2** |
| **8.2. Emotion (Anxiety)** | | | | | | | | | | |
| *3.1.1. Concerns about doing the bowel preparation* | **3-4, 3-5** | **2** | **2** | **2** | **0** | **0** | **2** | **0** | **0** | **2** |
| *3.1.3. Concerns about test invasiveness* | **3-4, 3-5** | **2** | **1** | **2** | **0** | **0** | **0** | **0** | **0** | **2** |
| *3.1.4. Shame and embarrassment* | **3-4, 3-5** | **1** | **0** | **1** | **1** | **0** | **0** | **0** | **0** | **2** |
| *3.1.5. Concerns about availability and necessity of sedation* | **3-4, 3-5** | **2** | **1** | **1** | **0** | **0** | **0** | **0** | **0** | **2** |
| *3.1.6. Concerns about perforation and procedural risks* | **3-4, 3-5** | **2** | **2** | **2** | **0** | **0** | **0** | **0** | **0** | **2** |
| *3.3.1. Anxiety* | **1-5** | **0** | **1** | **0** | **0** | **0** | **0** | **0** | **2** | **2** |
| *3.3.3. Avoidance* | **2-4** | **0** | **1** | **0** | **0** | **0** | **0** | **0** | **2** | **2** |
| **8.3. Emotion (Affect)** | | | | | | | | | | |
| *-* | **-** | - | **-** | **-** | **-** | **-** | **-** | **-** | **-** | **-** |
| **8.4. Emotion (Stress)** | | | | | | | | | | |
| *-* | **-** | - | **-** | **-** | **-** | **-** | **-** | **-** | **-** | **-** |
| **8.5. Emotion (Depression)** | | | | | | | | | | |
| *-* | **-** | - | **-** | **-** | **-** | **-** | **-** | **-** | **-** | **-** |
| **8.6. Emotion (Positive/negative affect)** | | | | | | | | | | |
| *-* | **-** | - | **-** | **-** | **-** | **-** | **-** | **-** | **-** | **-** |
| **8.7. Emotion (Burn-out)** | | | | | | | | | | |
| *-* | **-** | - | **-** | **-** | **-** | **-** | **-** | **-** | **-** | **-** |
| **9.1. Behavioural regulation (Self-monitoring)** | | | | | | | | | | |
| - | **-** | **-** | **-** | **-** | **-** | **-** | **-** | **-** | **-** | **-** |
| **9.2. Behavioural regulation (Breaking habit)** | | | | | | | | | | |
| - | **-** | **-** | **-** | **-** | **-** | **-** | **-** | **-** | **-** | **-** |
| **9.3. Behavioural regulation (Action planning)** | | | | | | | | | | |
|  | **-** | - | **-** | **-** | **-** | **-** | **-** | **-** | **-** | **-** |

| **ESM Table 6.** Allocation of BCTs to individual barriers | | | | | | | | | | | | | | | | | | | | | | | | | |
| --- | --- | --- | --- | --- | --- | --- | --- | --- | --- | --- | --- | --- | --- | --- | --- | --- | --- | --- | --- | --- | --- | --- | --- | --- | --- |
| **Factors identified in Kerrison et al., 2021 & 2022** | **1.2. PS** | | **3.1. & 3.2 SS** | | **4.1 IoHtPB** | | **5.1. IaHC** | | | **5.2. SoC** | | **6.1. DotB^a^** | | **6.2. SC** | | | **6.3. IaOA** | | **9.2 P&Cs** | | **11.2 RNE** | | **15.1 VPAC** | | **15.4 ST** |
| **1.1. Knowledge (Knowledge of condition / scientific rationale)** | | | | | | | | | | | | | | | | | | | | | | | | | |
| *3.2.1. Lack of understanding that bowel cancer can be asymptomatic and the test is looking for invisible traces of blood* | **No** | | **No** | | **No** | | **Yes** | | | **Yes** | | **No** | | **No** | | | **No** | | **No** | | **No** | | **No** | | **No** |
| *3.5.4.2. Belief that cancer is a treatable disease* | **No** | | **No** | | **No** | | **Yes** | | | **Yes** | | **No** | | **No** | | | **No** | | **No** | | **No** | | **No** | | **No** |
| **1.2. Knowledge (Procedural knowledge)** | | | | | | | | | | | | | | | | | | | | | | | | | |
| *1.3.1 Hearing other people’s experiences with colonoscopy* | **No** | | **No** | | **No** | | **No** | | | **No** | | **Yes** | | **No** | | | **No** | | **No** | | **No** | | **No** | | **No** |
| *1.3.2. Personal and family experiences with colonoscopy* | **No** | | **No** | | **No** | | **No** | | | **No** | | **No** | | **No** | | | **No** | | **No** | | **No** | | **No** | | **No** |
| *3.2.2. Lack of awareness and understanding of colonoscopy procedure* | **No** | | **No** | | **Yes** | | **No** | | | **Yes** | | **Yes** | | **No** | | | **No** | | **No** | | **No** | | **No** | | **No** |
| *4.2.3. Previous personal experiences with colonoscopy and other medical investigations* | **No** | | **No** | | **No** | | **No** | | | **No** | | **No** | | **No** | | | **No** | | **No** | | **No** | | **No** | | **No** |
| **2.3. Beliefs about capabilities (Self-efficacy)** | | | | | | | | | | | | | | | | | | | | | | | | | |
| *4.2.2. Existing health conditions interfering with ability to complete procedure or bowel prep* | **No** | | **Yes** | | **Yes** | | **No** | | | **No** | | **Yes** | | **No** | | | **No** | | **No** | | **No** | | **Yes** | | **Yes** |
| *4.2.1. Recent Colonoscopy* | **No** | | **No** | | **No** | | **No** | | | **No** | | **No** | | **No** | | | **No** | | **No** | | **No** | | **No** | | **No** |
| **3.1. Beliefs about consequences (Beliefs)** | | | | | | | | | | | | | | | | | | | | | | | | | |
| *1.2.4. Fatalistic beliefs* | **No** | | **No** | | **No** | | **Yes** | | | **Yes** | | **Yes** | | **No** | | | **No** | | **No** | | **No** | | **No** | | **No** |
| *1.2.7. Lack of trust in Western Medicine* | **No** | | **No** | | **No** | | **Yes** | | | **No** | | **Yes** | | **No** | | | **No** | | **No** | | **No** | | **No** | | **No** |
| *3.5.6. Perceived importance of screening* | **No** | | **No** | | **No** | | **Yes** | | | **Yes** | | **Yes** | | **No** | | | **No** | | **Yes** | | **No** | | **No** | | **No** |
| *3.9.6. The role of God in determining the future* | **No** | | **No** | | **No** | | **Yes** | | | **Yes** | | **Yes** | | **No** | | | **No** | | **No** | | **No** | | **No** | | **No** |
| **5.2. Environmental Context and Resources (Resources / material resources)** | | | | | | | | | | | | | | | | | | | | | | | | | |
| *2.1.1. Language barriers* | **No** | | **Yes** | | **No** | | **No** | | | **No** | | **No** | | **No** | | | **No** | | **No** | | **No** | | **No** | | **No** |
| **5.5. Environmental Context and Resources (Person × environment interaction)** | | | | | | | | | | | | | | | | | | | | | | | | | |
| *4.2.4. Existing health conditions as a competing priority* | **No** | | **No** | | **No** | | **No** | | | **No** | | **No** | | **No** | | | **No** | | **No** | | **No** | | **No** | | **No** |
| **5.6. Environmental Context and Resources (Barriers and facilitators)** | | | | | | | | | | | | | | | | | | | | | | | | | |
| *2.2.1. Transport / travel* | **Yes** | | **Yes** | | **Yes** | | **No** | | | **No** | | **No** | | **No** | | | **No** | | **No** | | **No** | | **No** | | **No** |
| *2.2.2. Travelling / on holiday* | **No** | | **No** | | **No** | | **No** | | | **No** | | **No** | | **No** | | | **No** | | **No** | | **No** | | **No** | | **No** |
| *2.2.3. Family, work and religious commitments* | **No** | | **No** | | **No** | | **No** | | | **No** | | **No** | | **No** | | | **No** | | **No** | | **No** | | **No** | | **No** |
| *2.2.4. Lack of car parking* | **Yes** | | **Yes** | | **No** | | **No** | | | **No** | | **No** | | **No** | | | **No** | | **No** | | **No** | | **No** | | **No** |
| *2.2.5. Indirect costs* | **No** | | **Yes** | | **No** | | **No** | | | **No** | | **No** | | **No** | | | **No** | | **No** | | **No** | | **No** | | **No** |
| **6.3. Skills (Ability)** | | | | | | | | | | | | | | | | | | | | | | | | | |
| *3.4.2. Low health literacy* | **No** | | **Yes** | | **No** | | **Yes** | | | **Yes** | | **No** | | **No** | | | **No** | | **No** | | **No** | | **No** | | **No** |
| **7.2. Social Influences (Social norms)** | | | | | | | | | | | | | | | | | | | | | | | | | |
| 1*.2.1. Unable to have a male practitioner* | **No** | | **No** | | **No** | | **No** | | | **No** | | **No** | | **No** | | | **No** | | **No** | | **No** | | **No** | | **No** |
| *1.2.2. Colonoscopy, colon and rectum ‘culturally taboo’ topics* | **No** | | **Yes** | | **No** | | **No** | | | **No** | | **Yes** | | **Yes** | | | **Yes** | | **No** | | **No** | | **No** | | **No** |
| 1*.2.5. Unable to accept blood products* | **No** | | **No** | | **No** | | **No** | | | **No** | | **Yes** | | **No** | | | **No** | | **No** | | **No** | | **No** | | **No** |
| **7.5. Social Influences (Group norms**) | | | | | | | | | | | | | | | | | | | | | | | | | |
| *1.2.3. Gender and engagement with healthcare* | **No** | | **No** | | **No** | | **No** | | | **No** | | **Yes** | | **Yes** | | | **Yes** | | **No** | | **No** | | **No** | | **No** |
| **7.6. Social Influences (Social support)** | | | | | | | | | | | | | | | | | | | | | | | | | |
| *1.4.1. Reliance on family and friends as unofficial interpreters* | **No** | | **Yes** | | **No** | | **No** | | | **No** | | **No** | | **No** | | | **No** | | **No** | | **No** | | **No** | | **No** |
| *1.4.2. Reliance on family for travel & transport* | **No** | | **Yes** | | **No** | | **No** | | | **No** | | **No** | | **No** | | | **No** | | **No** | | **No** | | **No** | | **No** |
| *1.4.3. Reliance on family for emotional support* | **No** | | **Yes** | | **No** | | **No** | | | **No** | | **No** | | **No** | | | **No** | | **No** | | **No** | | **No** | | **No** |
| *3.9.4. Shared decision making and family influenced participation* | **No** | | **Yes** | | **No** | | **No** | | | **No** | | **No** | | **No** | | | **No** | | **No** | | **No** | | **No** | | **No** |
| **7.7. Social Influences (Power)** | | | | | | | | | | | | | | | | | | | | | | | | | |
| *3.9.3. Reliance on medical professional / authority* | **No** | | **Yes** | | **No** | | **No** | | | **No** | | **No** | | **No** | | | **Yes** | | **No** | | **No** | | **No** | | **No** |
| **8.1. Emotion (Fear)** | | | | | | | | | | | | | | | | | | | | | | | | | |
| *3.1.2. Fear of pain and discomfort* | **No** | | **Yes** | | **No** | | **No** | | | **No** | | **Yes** | | **No** | | | **No** | | **No** | | **Yes** | | **Yes** | | **Yes** |
| *3.5.4.1. Fear of cancer* | **No** | | **Yes** | | **No** | | **No** | | | **No** | | **Yes** | | **No** | | | **No** | | **No** | | **Yes** | | **No** | | **Yes** |
| **8.2. Emotion (Anxiety)** | | | | | | | | | | | | | | | | | | | | | | | | | |
| *3.1.1. Concerns about doing the bowel preparation* | **No** | | **No** | | **Yes** | | **No** | | | **No** | | **Yes** | | **No** | | | **No** | | **No** | | **Yes** | | **Yes** | | **Yes** |
| *3.1.3. Concerns about test invasiveness* | **No** | | **No** | | **No** | | **No** | | | **No** | | **Yes** | | **No** | | | **No** | | **No** | | **Yes** | | **No** | | **Yes** |
| *3.1.4. Shame and embarrassment* | **No** | | **No** | | **No** | | **No** | | | **No** | | **Yes** | | **No** | | | **Yes** | | **No** | | **Yes** | | **No** | | **Yes** |
| *3.1.5. Concerns about availability and necessity of sedation* | **No** | | **No** | | **No** | | **No** | | | **No** | | **No** | | **No** | | | **No** | | **No** | | **Yes** | | **No** | | **No** |
| *3.1.6. Concerns about perforation and procedural risks* | **No** | | **No** | | **No** | | **No** | | | **No** | | **No** | | **No** | | | **No** | | **No** | | **Yes** | | **No** | | **No** |
| *3.3.1. Anxiety* | **No** | | **Yes** | | **No** | | **No** | | | **No** | | **No** | | **No** | | | **No** | | **No** | | **Yes** | | **Yes** | | **Yes** |
| *3.3.3. Avoidance* | **No** | | **Yes** | | **No** | | **No** | | | **No** | | **No** | | **No** | | | **No** | | **No** | | **Yes** | | **No** | | **Yes** |
| **Orange = Evidence to support effectiveness of BCT to target domain, but BCT does not obviously apply to barrier identified**  **Red = No BCTs identified**  *** = Exclude BCT – Unethical in context / Against ethos of informed decision making?**  **^a^ Modelling could be used to address many of the barriers; however, could not be incorporated as part of a letter (would need to be leaflet or video)** | | | | | | | | | | | | | | | | | | | | | | | | | |
| **ESM Table 7.** TDF-BCT Matrix | | | | | | | | | | | | | | | | | | | | | | | |  |  |
| **Factors identified in Kerrison et al., 2021 & 2022** | **1.2. PS** | **3.1. & 3.2 SS** | | **4.1 IoHtPB** | | **5.1. IaHC** | | **5.2. SoC** | **6.1. DotB^a^** | | **6.2. SC** | | **6.3. IaOA** | | **9.2 P&Cs** | **11.2 RNE** | | **15.1 VPAC** | | **15.4 ST** | | **Letter**  **Yes/No** | |  |  |
| **1.1. Knowledge (Knowledge of condition / scientific rationale)** | | | | | | | | | | | | | | | | | | | | | | | |  |  |
| *3.2.1. Lack of understanding that bowel cancer can be asymptomatic and the test is looking for invisible traces of blood* |  |  | |  | | **X** | | **X** |  | |  | |  | |  |  | |  | |  | | **Yes** | |  |  |
| *3.5.4.2. Belief that cancer is a treatable disease* |  |  | |  | | **X** | | **X** |  | |  | |  | |  |  | |  | |  | | **Yes** | |  |  |
| **1.2. Knowledge (Procedural knowledge)** | | | | | | | | | | | | | | | | | | | | | | | |  |  |
| *1.3.1 Hearing other people’s experiences with colonoscopy* |  |  | |  | |  | |  | **X** | |  | |  | |  |  | |  | |  | | **Yes** | |  |  |
| *3.2.2. Lack of awareness and understanding of colonoscopy procedure* |  |  | | **X** | |  | |  | **X** | |  | |  | |  |  | |  | |  | | **Yes** | |  |  |
| **2.3. Beliefs about capabilities (Self-efficacy)** | | | | | | | | | | | | | | | | | | | | | | | |  |  |
| *4.2.2. Existing health conditions interfering with ability to complete procedure or bowel prep* |  | **X** | | **X** | |  | |  | **X** | |  | |  | |  |  | | **X** | | **X** | | **Yes** | |  |  |
| **3.1. Beliefs about consequences (Beliefs)** | | | | | | | | | | | | | | | | | | | | | | | |  |  |
| *1.2.4. Fatalistic beliefs* |  |  | |  | | **X** | | **X** | **X** | |  | |  | |  |  | |  | |  | | **Yes** | |  |  |
| *1.2.7. Lack of trust in Western Medicine* |  |  | |  | | **X** | |  | **X** | |  | |  | |  |  | |  | |  | | **Yes** | |  |  |
| *3.5.6. Perceived importance of screening* |  |  | |  | | **X** | | **X** | **X** | |  | |  | | **X** |  | |  | |  | | **Yes** | |  |  |
| *3.9.6. The role of God in determining the future* |  |  | |  | | **X** | | **X** | **X** | |  | |  | |  |  | |  | |  | | **Yes** | |  |  |
| **5.2. Environmental Context and Resources (Resources / material resources)** | | | | | | | | | | | | | | | | | | | | | | | |  |  |
| *2.1.1. Language barriers* |  | **X** | |  | |  | |  |  | |  | |  | |  |  | |  | |  | | **Yes** | |  |  |
| **5.6. Environmental Context and Resources (Barriers and facilitators)** | | | | | | | | | | | | | | | | | | | | | | | |  |  |
| *2.2.1. Transport / travel* | **X** | **X** | |  | |  | |  |  | |  | |  | |  |  | |  | |  | | **Yes** | |  |  |
| *2.2.4. Lack of car parking* | **X** | **X** | |  | |  | |  |  | |  | |  | |  |  | |  | |  | | **Yes** | |  |  |
| *2.2.5. Indirect costs* |  | **X** | |  | |  | |  |  | |  | |  | |  |  | |  | |  | | **Yes** | |  |  |
| **6.3. Skills (Ability)** | | | | | | | | | | | | | | | | | | | | | | | |  |  |
| *3.4.2. Low health literacy* |  | **X** | |  | | **X** | | **X** |  | |  | |  | |  |  | |  | |  | | **Yes** | |  |  |
| **7.2. Social Influences (Social norms)** | | | | | | | | | | | | | | | | | | | | | | | |  |  |
| *1.2.2. Colonoscopy, colon and rectum ‘culturally taboo’ topics* |  | **X** | |  | |  | |  | **X** | | **X** | | **X** | |  |  | |  | |  | | **Yes** | |  |  |
| 1*.2.5. Unable to accept blood products* |  |  | |  | |  | |  | **X** | |  | |  | |  |  | |  | |  | | **Yes** | |  |  |
| **7.5. Social Influences (Group norms**) | | | | | | | | | | | | | | | | | | | | | | | |  |  |
| *1.2.3. Gender and engagement with healthcare* |  |  | |  | |  | |  | **X** | | **X** | | **X** | |  |  | |  | |  | | **Yes** | |  |  |
| **7.6. Social Influences (Social support)** 0.70 | | | | | | | | | | | | | | | | | | | | | | | |  |  |
| *1.4.1. Reliance on family and friends as unofficial interpreters* |  | **X** | |  | |  | |  |  | |  | |  | |  |  | |  | |  | | **Yes** | |  |  |
| *1.4.2. Reliance on family for travel & transport* |  | **X** | |  | |  | |  |  | |  | |  | |  |  | |  | |  | | **Yes** | |  |  |
| *1.4.3. Reliance on family for emotional support* |  | **X** | |  | |  | |  |  | |  | |  | |  |  | |  | |  | | **Yes** | |  |  |
| *3.9.4. Shared decision making and family influenced participation* |  | **X** | |  | |  | |  |  | |  | |  | |  |  | |  | |  | | **Yes** | |  |  |
| **7.7. Social Influences (Power)** | | | | | | | | | | | | | | | | | | | | | | | |  |  |
| *3.9.3. Reliance on medical professional / authority* |  | **X** | |  | |  | |  |  | |  | | **X** | |  |  | |  | |  | | **Yes** | |  |  |
| **8.1. Emotion (Fear)** 0.81 | | | | | | | | | | | | | | | | | | | | | | | |  |  |
| *3.1.2. Fear of pain and discomfort* |  | **X** | |  | |  | |  | **X** | |  | |  | |  | **X** | | **X** | | **X** | | **Yes** | |  |  |
| *3.5.4.1. Fear of cancer* |  | **X** | |  | |  | |  | **X** | |  | |  | |  | **X** | |  | | **X** | | **Yes** | |  |  |
| **8.2. Emotion (Anxiety)** | | | | | | | | | | | | | | | | | | | | | | | |  |  |
| *3.1.1. Concerns about doing the bowel preparation* |  |  | | **X** | |  | |  | **X** | |  | |  | |  | **X** | | **X** | | **X** | | **Yes** | |  |  |
| *3.1.3. Concerns about test invasiveness* |  |  | |  | |  | |  | **X** | |  | |  | |  | **X** | |  | | **X** | | **Yes** | |  |  |
| *3.1.4. Shame and embarrassment* |  |  | |  | |  | |  | **X** | |  | | **X** | |  | **X** | |  | | **X** | | **Yes** | |  |  |
| *3.3.1. Anxiety* |  | **X** | |  | |  | |  |  | |  | |  | |  | **X** | | **X** | | **X** | | **Yes** | |  |  |
| *3.3.3. Avoidance* |  | **X** | |  | |  | |  |  | |  | |  | |  | **X** | |  | | **X** | | **Yes** | |  |  |

| **ESM Table 8.** Overview of selected barriers and corresponding survey items | |
| --- | --- |
| **Barrier** | **Survey item** |
| **Psychological variables** | |
| **1.1. Knowledge (Knowledge of condition / scientific rationale)** | |
| *Lack of understanding that bowel cancer can be asymptomatic and the test is looking for invisible traces of blood* | Bowel cancer screening is only useful for people with symptoms |
| *Belief that cancer is a treatable disease* | Bowel cancer is not a treatable disease |
| **1.2. Knowledge (Procedural knowledge)** | |
| *Hearing other people’s experiences with colonoscopy* | Other people’s experiences with colonoscopy have put me off going for colonoscopy myself |
| *Lack of awareness and understanding of colonoscopy procedure* | I have a good understanding of what having a colonoscopy would involve |
| **2.3. Beliefs about capabilities (Self-efficacy)** | |
| *Existing health conditions interfering with ability to complete procedure or bowel prep* | I have existing health conditions that would prevent me from being able to have a colonoscopy |
| **3.1. Beliefs about consequences (Beliefs)** | |
| *Fatalistic beliefs* | Being diagnosed with bowel cancer is a death sentence |
| *Lack of trust in Western Medicine* | Western medicine is not effective at treating bowel cancer |
| *Perceived importance of screening* | It is important to take part in bowel cancer screening |
| *The role of God in determining the future* | It is God’s decision who lives and dies, medicine cannot change that |
| **5.2. Environmental Context and Resources (Resources / material resources)** | |
| *Language barriers* | I would need an interpreter to translate during the nurse appointment |
| **5.6. Environmental Context and Resources (Barriers and facilitators)** | |
| *Transport / travel* | I would find it difficult to travel to the appointment with the nurse |
| *Lack of car parking* | If I drove to the appointment, it would be difficult to find a car parking space at my local hospital |
| *Indirect costs* | Going to the appointment (e.g. parking, public transport, time off work, etc) would cost me a lot of money |
| **7.2. Social Influences (Social norms)** | |
| *Colonoscopy, colon and rectum ‘culturally taboo’ topics* | I would not be able to discuss this appointment with my friends and family, as this type of thing is very sensitive |
| *Unable to accept blood products* | I would be worried that a blood transfusion might be needed if I had a colonoscopy, and I am unwilling to have these on religious grounds |
| **7.5. Social Influences (Group norms**) | |
| *Gender and engagement with healthcare* | It is fine for women to have colonoscopy, but not men |
| **7.6. Social Influences (Social support)** 0.70 | |
| *Reliance on family and friends as unofficial interpreters* | I would need to take a friend or family member with me to translate what the nurse was saying |
| *Reliance on family for travel & transport* | I would need a friend or family member to take me to the nurse appointment |
| *Reliance on family for emotional support* | I would need to take a friend or family member with me to the nurse appointment for emotional support |
| *Shared decision making and family influenced participation* | I would only go to the nurse appointment if a friend or family member agreed it was in my best interest to attend |
| **7.7. Social Influences (Power)** | |
| *Reliance on medical professional / authority* | I would only go to the nurse appointment if my general practitioner told me I should go |
| **8.1. Emotion (Fear)** 0.81 | |
| *Fear of pain and discomfort* | I would be worried that the colonoscopy would be painful or uncomfortable |
| *Fear of cancer* | I would be scared that the colonoscopy would find cancer |
| **8.2. Emotion (Anxiety)** | |
| *Concerns about doing the bowel preparation* | I would be worried about doing the bowel preparation / drinking powerful laxatives before the colonoscopy |
| *Concerns about test invasiveness* | I am worried about the invasive nature of colonoscopy |
| *Shame and embarrassment* | Going for colonoscopy would be shameful and / or embarrassing |
| *Anxiety* | I would feel anxious about going to the nurse appointment |
| *Avoidance* | I would prefer not to know whether I had bowel cancer |
| **Demographic variables** | |
| *Age* | What is your age in years? |
| *Gender* | Which of the following best describes you? |
| *Long-term conditions* | Do you have any long-term conditions? |
| *Disabilities* | Do you have any disabilities? |
| *Existing mental health conditions* | Do you have an existing mental health condition? |
| *Main language* | What is your main language? |
| *Ethnicity* | Which of these best describes your ethnicity? |
| *Education* | What is the highest level of education qualification you have obtained? |

| **ESM Table 9.** CHERRIES Checklist | | |
| --- | --- | --- |
| **Item** | **Description** | **Page number** |
| **Design** | | |
| Describe survey design | Describe target population, sample frame. Is the sample a convenience sample? (In “open” surveys this is most likely.) | 5 |
| **IRB (Institutional Review Board) approval and informed consent process** | | |
| IRB approval | Mention whether the study has been approved by an IRB. | 11 |
| Informed consent | Describe the informed consent process. Where were the participants told the length of time of the survey, which data were stored and where and for how long, who the investigator was, and the purpose of the study? | 11 |
| Data protection | If any personal information was collected or stored, describe what mechanisms were used to protect unauthorized access. | 11 |
| **Development and pre-testing** | | |
| Development and testing | State how the survey was developed, including whether the usability and technical functionality of the electronic questionnaire had been tested before | 6-10 |
| **Recruitment process and description of the sample having access to the questionnaire** | | |
| Open survey versus closed survey | An “open survey” is a survey open for each visitor of a site, while a closed survey is only open to a sample which the investigator knows (password-protected survey). | 4 |
| Contact mode | Indicate whether or not the initial contact with the potential participants was made on the Internet. (Investigators may also send out questionnaires by mail and allow for Web-based data entry.) | 5 |
| Advertising the survey | How/where was the survey announced or advertised? Some examples are offline media (newspapers), or online (mailing lists – If yes, which ones?) or banner ads (Where were these banner ads posted and what did they look like?). It is important to know the wording of the announcement as it will heavily influence who chooses to participate. Ideally the survey announcement should be published as an appendix. | 5 |
| **Survey administration** | | |
| Web/E-mail | State the type of e-survey (eg, one posted on a Web site, or one sent out through e-mail). If it is an e-mail survey, were the responses entered manually into a database, or was there an automatic method for capturing responses? | 5 |
| Context | Describe the Web site (for mailing list/newsgroup) in which the survey was posted. What is the Web site about, who is visiting it, what are visitors normally looking for? Discuss to what degree the content of the Web site could pre-select the sample or influence the results. For example, a survey about vaccination on a anti-immunization Web site will have different results from a Web survey conducted on a government Web site | 5 |
| Mandatory/voluntary | Was it a mandatory survey to be filled in by every visitor who wanted to enter the Web site, or was it a voluntary survey? | 5 |
| Incentives | Were any incentives offered (eg, monetary, prizes, or non-monetary incentives such as an offer to provide the survey results)? | 5 |
| Time/Date | In what timeframe were the data collected? | 10 |
| Randomization of items or questionnaires | To prevent biases items can be randomized or alternated. | 5 |
| Adaptive questioning | Use adaptive questioning (certain items, or only conditionally displayed based on responses to other items) to reduce number and complexity of the questions. | NA |
| Number of Items | What was the number of questionnaire items per page? The number of items is an important factor for the completion rate. | 5, 9 |
| Number of screens (pages) | Over how many pages was the questionnaire distributed? The number of items is an important factor for the completion rate. | 9, 10 |
| Completeness check | It is technically possible to do consistency or completeness checks before the questionnaire is submitted. Was this done, and if “yes”, how (usually JAVAScript)? An alternative is to check for completeness after the questionnaire has been submitted (and highlight mandatory items). If this has been done, it should be reported. All items should provide a non-response option such as “not applicable” or “rather not say”, and selection of one response option should be enforced. | 10, 11 |
| Review step | State whether respondents were able to review and change their answers (eg, through a Back button or a Review step which displays a summary of the responses and asks the respondents if they are correct). | 10 |
| **Response rates** | | |
| Unique site visitor | If you provide view rates or participation rates, you need to define how you determined a unique visitor. There are different techniques available, based on IP addresses or cookies or both. | 10 |
| View rate (Ratio of unique survey visitors/unique site visitors) | Requires counting unique visitors to the first page of the survey, divided by the number of unique site visitors (not page views!). It is not unusual to have view rates of less than 0.1 % if the survey is voluntary. | 11 |
| Participation rate (Ratio of unique visitors who agreed to participate/unique first survey page visitors) | Count the unique number of people who filled in the first survey page (or agreed to participate, for example by checking a checkbox), divided by visitors who visit the first page of the survey (or the informed consents page, if present). This can also be called “recruitment” rate. | 11, 12 |
| Completion rate (Ratio of users who finished the survey/users who agreed to participate) | The number of people submitting the last questionnaire page, divided by the number of people who agreed to participate (or submitted the first survey page). This is only relevant if there is a separate “informed consent” page or if the survey goes over several pages. This is a measure for attrition. Note that “completion” can involve leaving questionnaire items blank. This is not a measure for how completely questionnaires were filled in. (If you need a measure for this, use the word “completeness rate”.) | 11, 12 |
| **Preventing multiple entries from the same individual** | | |
| Cookies used | Indicate whether cookies were used to assign a unique user identifier to each client computer. If so, mention the page on which the cookie was set and read, and how long the cookie was valid. Were duplicate entries avoided by preventing users access to the survey twice; or were duplicate database entries having the same user ID eliminated before analysis? In the latter case, which entries were kept for analysis (eg, the first entry or the most recent)? | NA |
| IP check | Indicate whether the IP address of the client computer was used to identify potential duplicate entries from the same user. If so, mention the period of time for which no two entries from the same IP address were allowed (eg, 24 hours). Were duplicate entries avoided by preventing users with the same IP address access to the survey twice; or were duplicate database entries having the same IP address within a given period of time eliminated before analysis? If the latter, which entries were kept for analysis (eg, the first entry or the most recent)? | NA |
| Log file analysis | Indicate whether other techniques to analyze the log file for identification of multiple entries were used. If so, please describe. | NA |
| Registration | In “closed” (non-open) surveys, users need to login first and it is easier to prevent duplicate entries from the same user. Describe how this was done. For example, was the survey never displayed a second time once the user had filled it in, or was the username stored together with the survey results and later eliminated? If the latter, which entries were kept for analysis (eg, the first entry or the most recent)? | 11 |
| **Analysis** | | |
| Handling of incomplete questionnaires | Were only completed questionnaires analyzed? Were questionnaires which terminated early (where, for example, users did not go through all questionnaire pages) also analyzed? | 10, 12 |
| Questionnaires submitted with an atypical timestamp | Some investigators may measure the time people needed to fill in a questionnaire and exclude questionnaires that were submitted too soon. Specify the timeframe that was used as a cut-off point, and describe how this point was determined. | NA |
| Statistical correction | Indicate whether any methods such as weighting of items or propensity scores have been used to adjust for the non-representative sample; if so, please describe the methods. | 10 |

| **ESM Table 10.** Predictors of colonoscopy intentions: Mean scores, proportions and unadjusted and adjusted Odds Ratios – results from univariate and multivariate binary logistic regression with ethnic minority groups (n=48) | | | |
| --- | --- | --- | --- |
|  | **All other responses**  **(Range 1-5)** | **Yes, definitely**  **(Range 1-5)** | **OR (95%CI)** |
| **Psychological variables** | | | |
| **1.1. Knowledge (Knowledge of condition / scientific rationale)** | | | |
| *3.2.1. Lack of understanding that bowel cancer can be asymptomatic and the test is looking for invisible traces of blood* | 2.63 | 1.85 | 0.45 (0.20, 1.00) |
| *3.5.4.2. Belief that cancer is a treatable disease* | 2.50 | 1.90 | 0.51 (0.23, 1.16) |
| **1.2. Knowledge (Procedural knowledge)** | | | |
| *1.3.1 Hearing other people’s experiences with colonoscopy* | 2.13 | 1.90 | 0.56 (0.16, 1.94) |
| *3.2.2. Lack of awareness and understanding of colonoscopy procedure* | 2.25 | 2.33 | 1.09 (0.49, 2.44) |
| **2.3. Beliefs about capabilities (Self-efficacy)** | | | |
| *4.2.2. Existing health conditions interfering with ability to complete procedure or bowel prep* | 1.75 | 1.55 | 0.59 (0.18, 2.01) |
| **3.1. Beliefs about consequences (Beliefs)** | | | |
| *1.2.4. Fatalistic beliefs* | 2.63 | 2.32 | 0.58 (0.20, 1.68) |
| *1.2.7. Lack of trust in Western Medicine* | 2.63 | 1.88 | **0.29 (0.10, 0.89)*** |
| *3.5.6. Perceived importance of screening* | 2.50 | 1.25 | **0.05 (0.01, 0.40)**** |
| *3.9.6. The role of God in determining the future* | 2.38 | 1.87 | 0.72 (0.40, 1.30) |
| **5.2. Environmental Context and Resources (Resources / material resources)** | | | |
| *2.1.1. Language barriers* | 1.63 | 1.35 | 0.41 (0.10, 1.58) |
| **5.6. Environmental Context and Resources (Barriers and facilitators)** | | | |
| *2.2.1. Transport / travel* | 2.75 | 1.78 | **0.28 (0.11, 0.75)*** |
| *2.2.4. Lack of car parking* | 3.13 | 3.00 | 0.93 (0.51, 1.68) |
| *2.2.5. Indirect costs* | 2.50 | 2.28 | 0.80 (0.38, 1.68) |
| **7.2. Social Influences (Social norms)** | | | |
| *1.2.2. Colonoscopy, colon and rectum ‘culturally taboo’ topics* | 2.88 | 2.05 | **0.40 (0.17, 0.94)*** |
| 1*.2.5. Unable to accept blood products* | 1.88 | 1.50 | 0.54 (0.21, 1.39) |
| **7.5. Social Influences (Group norms**) | | | |
| *1.2.3. Gender and engagement with healthcare* | 1.75 | 1.40 | 0.51 (0.18, 1.41) |
| **7.6. Social Influences (Social support)** 0.70 | | | |
| *1.4.1. Reliance on family and friends as unofficial interpreters* | 2.00 | 1.45 | 0.48 (0.20, 1.13) |
| *1.4.2. Reliance on family for travel & transport* | 2.63 | 2.30 | 0.78 (0.41, 1.50) |
| *1.4.3. Reliance on family for emotional support* | 2.75 | 2.73 | 0.98 (0.53, 1.83) |
| *3.9.4. Shared decision making and family influenced participation* | 2.50 | 1.65 | **0.44 (0.20, 0.94)*** |
| **7.7. Social Influences (Power)** | | | |
| *3.9.3. Reliance on medical professional / authority* | 2.75 | 1.72 | **0.39 (0.18, 0.85)*** |
| **8.1. Emotion (Fear)** | | | |
| *3.1.2. Fear of pain and discomfort* | 3.62 | 3.45 | 0.83 (0.37, 1.86) |
| *3.5.4.1. Fear of cancer* | 3.38 | 3.50 | 1.12 (0.55, 2.27) |
| **8.2. Emotion (Anxiety)** | | | |
| *3.1.1. Concerns about doing the bowel preparation* | 3.00 | 2.70 | 0.79 (0.39, 1.57) |
| *3.1.3. Concerns about test invasiveness* | 3.25 | 2.95 | 0.77 (0.37, 1.60) |
| *3.1.4. Shame and embarrassment* | 2.63 | 1.78 | **0.50 (0.25, 1.00)*** |
| *3.3.1. Anxiety* | 3.25 | 3.25 | 1.00 (0.52, 1.94) |
| *3.3.3. Avoidance* | 2.62 | 1.52 | **0.30 (0.12, 0.72)**** |
| **Demographic variables** | | | |
| **Age** | | | |
| *Years (continuous)* | 59.38 | 60.85 | 1.06 (0.91, 1.24) |
| **Gender** | | | |
| *Male* | 6 (75.0) | 19 (47.5) | - |
| *Female* | 2 (25.0) | 21 (52.5) | **-** |
| *Non-binary* | - | - | **-** |
| **Long term conditions** | | | |
| *No* | 7 (87.5) | 23 (57.5) | - |
| *Yes* | 1 (12.5) | 17 (42.5) | **-** |
| **Disability** | | | |
| *No* | 8 (100.00) | 35 (87.5) | - |
| *Yes* | - | 5 (12.5) | **-** |
| **Mental health** | | | |
| *No* | 6 (75.0) | 39 (97.5) | - |
| *Yes* | 2 (25.0) | 1 (2.5) | **-** |
| **Main language** | | | |
| *English* | 7 (87.5) | 36 (90.0) | - |
| *Other* | 1 (12.5) | 4 (10.0) | **-** |
| **Ethnicity** | | | |
| *White British / Irish* | 0 (0.00) | 0 (0.00) | - |
| *Any Other Ethnicity* | 8 (100.00) | 40 (100.00) | **-** |
| **Education** | | | |
| *<O-level or GCSE Grade A-C* | 0 (0.00) | 4 (10.00) | - |
| *>ONC / BTEC* | 10 (100.00) | 36 (90.00) | **-** |

| **ESM Table 11.** Reporting of Factorial Randomized Trials: Extension of the CONSORT2010 Statement | | | | |
| --- | --- | --- | --- | --- |
| Section | Item number | CONSORT 2010 statement checklist item | Extension for factorial trials | Page number |
| **Title and abstract** | | | | |
| Title | 1a | Identification as a randomized trial in the title | Identification as a factorial randomized trial in the title | 1 (Title page) |
| Abstract | 1b | Structured summary of trial design, methods, results, and conclusions (for specific guidance see CONSORT for abstracts) | See separate factorial checklist for abstracts | 1, 2 |
| **Introduction** | | | | |
| Background | 2a | Scientific background and explanation of rationale | Scientific background and rationale for using a factorial design, including whether an interaction is hypothesized | 3, 4 |
| Objectives | 2b | Specific objectives or hypotheses | Specific objectives or hypotheses and a statement of which treatment groups form the main comparisons | 4 |
| **Methods** | | | | |
| Trial design | 3a | Description of trial design (such as parallel, factorial) including allocation ratio | Description of the type of factorial trial (such as full or partial, number of factors, levels within each factor) and allocation ratio | 4, 15 |
| Change from the protocol | 3b | Important changes to methods after trial commencement(such as eligibility criteria), with reasons |  | NA |
| Participants | 4a | Eligibility criteria for participants | Eligibility criteria for each factor, noting any differences, if applicable | 5, 14 |
| Setting and location | 4b | Settings and locations where the data were collected |  | 4 |
| Interventions | 5 | The interventions for each group with sufficient details to allow replication, including how and when they were actually administered |  | 14, 15 |
| Outcomes | 6a | Completely defined pre-specified primary and secondary outcome measures, including how and when they were assessed |  | 15 |
| Changes to outcomes | 6b | Any changes to trial outcomes after the trial commenced, with reasons |  | NA |
| Sample size | 7a | How sample size was determined | How sample size was determined for each main comparison, including whether an interaction was assumed in the calculation | 16, 17 |
| Interim analyses and stopping guidelines | 7b | When applicable, explanation of any interim analyses and stopping guidelines | When applicable, explanation of any interim analyses and stopping guidelines, noting any differences across main comparisons and reasons for differences | NA |
| **Randomization** | | | | |
| Sequence generation | 8a | Method used to generate the random allocation sequence |  | 15 |
| Sequence generation | 8b | Type of randomization; details of any restriction (such as blocking and block size) | Type of randomization, details of any restriction (such as blocking and block size), and, if applicable, whether participants were randomized to factors at different timepoints | 15 |
| Allocation concealment mechanism | 9 | Mechanism used to implement the random allocation sequence (such as sequentially numbered containers), describing any steps taken to conceal the sequence until interventions were assigned |  | NA |
| Implementation | 10 | Who generated the random allocation sequence, who enrolled participants, and who assigned participants to interventions |  | 15 |
| Blinding | 11a | If done, who was blinded after assignment to interventions (for example, participants, care providers, those assessing outcomes) |  | 15 |
| Similarity of interventions | 11b | If relevant, description of the similarity of interventions |  | 15 |
| Statistical methods | 12a | Statistical methods used to compare groups for primary and secondary outcomes | Statistical methods used for each main comparison for primary and secondary outcomes, including: Whether the target treatment effect for each main comparison pertains to the effect in the presence or absence of other factors  Approach to analysis, such as factorial or multiarm  How the approach was chosen, such as prespecified or based on estimated interaction If factorial approach was used, whether factors were adjusted for each other  If applicable, how nonconcurrent recruitment to factors was handled Method(s)used to evaluate statistical interaction(s) | 16 |
| Additional analyses | 12b | Methods for additional analyses, such as subgroup analyses and adjusted analyses |  | 16 |
| **Results** | | | | |
| Participant flow (a diagram is strongly recommended) | 13a | For each group, the numbers of participants who were randomly assigned, received intended treatment, and were analyzed for the primary outcome | For each main comparison, the number of participants who were randomly assigned, received intended treatment, and were analyzed for the primary outcome | 18, 38 |
| Losses and exclusions | 13b | For each group, losses and exclusions after randomization, together with reasons | For each main comparison, losses and exclusions after randomization, together with reasons | 17, 38 |
| Recruitment | 14a | Dates defining the periods of recruitment and follow-up | Dates defining the periods of recruitment and follow-up for each factor, noting any differences, with reasons | 17, 38 |
| Trial end | 14b | Why the trial ended or was stopped |  | NA |
| Baeline data | 15 | A table showing baseline demographic and clinical characteristics for each group | A table showing baseline demographic and clinical characteristics for each main comparison | 32 |
| Numbers analysed | 16 | For each group, the number of participants (denominator)included in each analysis and whether the analysis was by original assigned groups | For each main comparison, the number of participants (denominator)included in each analysis and whether the analysis was by original assigned groups | 33 |
| Outcomes and estimation | 17a | For each primary and secondary outcome, results for each group and the estimated effect size and its precision (suchas95%CI) | For each primary and secondary outcome, results for each main comparison, the estimated effect size, and its precision (suchas95%CI)  For each primary outcome, the estimated interaction effect and its precision  If done, the estimated interaction effects and precision for secondary outcomes | 33-35 |
| Binary outcomes | 17b | For binary outcomes, presentation of both absolute and relative effect sizes is recommended |  | 33-35 |
| Ancillary analyses | 18a | Results of any other analyses performed, including subgroup analyses and adjusted analyses, distinguishing prespecified from exploratory |  | 19 |
| Additional data summaries | 18b |  | Participant flow, losses and exclusions, baseline data, and outcome data (including primary and secondary outcomes, harms, and adherence) presented by treatment groups | 18, 38 |
| Harms | 19 | All important harms or unintended effects in each group (for specific guidance see CONSORT for harms) | All important harms or unintended effects for each main comparison | MA |
| **Discussion** | | | | |
| Limitations | 20 | Trial limitations, addressing sources of potential bias, imprecision, and, if relevant, multiplicity of analyses |  | 22, 23 |
| Generalisability | 21 | Generalizability(external validity, applicability) of the trial findings |  | 22, 23 |
| Interpretation | 22 | Interpretation consistent with results, balancing benefits and harms, and considering other relevant evidence |  | 20-23 |
| **Other information** | | | | |
| Registration | 23 | Registration number and name of trial registry |  | NA |
| Protocol | 24 | Where the full trial protocol can be accessed, if available |  | NA |
| Funding | 25 | Sources of funding and other support (such as supply of drugs), role of funders |  | 2 (Title page) |

| **ESM Table 12.** Effectiveness of BCTs to modify intentions: number, proportions and unadjusted and adjusted Odds Ratios – results from the univariate and multivariate binary logistic regression analyses (ethnic minority groups only) (n=77) | | | |
| --- | --- | --- | --- |
|  | **All other responses**  **(Range 1-5)** | **Yes, definitely**  **(Range 1-5)** | **OR (95%CI)** |
|  | | | |
| BCT 1. Reduce negative emotions (off) | 13 (27.7) | 34 (72.3) | 1.00 |
| BCT 1. Reduce negative emotions (on) | 3 (10.0) | 27 (90.0) | 3.44 (0.89, 13.32) |
| BCT 2. Information about health consequences (off) | 10 (25.6) | 29 (74.4) | 1.00 |
| BCT 2. Information about health consequences (on) | 6 (15.8) | 32 (84.2) | 1.84 (0.59, 5.69) |
| BCT 3. Social support (unspecified) (off) | 9 (20.9) | 34 (79.1) | 1.00 |
| BCT 3. Social support (unspecified) (on) | 7 (20.6) | 27 (79.4) | 1.02 (0.34, 3.10) |
| BCT 4. Social support (practical) (off) | 6 (20.0) | 24 (80.0) | 1.00 |
| BCT 4. Social support (practical) (on) | 10 (21.3) | 37 (78.7) | 0.93 (0.30, 2.88) |
| BCT 5. Reduce negative emotions (off) | 6 (19.4) | 36 (78.3) | 1.00 |
| BCT 5. Reduce negative emotions (on) | 10 (21.7) | 36 (78.3) | 0.86 (0.28, 2.68) |
| BCT 6. Reduce negative emotions (off) | 10 (23.3) | 33 (76.7) | 1.00 |
| BCT 6. Reduce negative emotions (on) | 6 (17.6) | 28 (82.4) | 1.41 (0.46, 4.38) |
| BCT 7. Social support (practical) (off) | 9 (27.3) | 24 (72.7) | 1.00 |
| BCT 7. Social support (practical) (on) | 7 (15.9) | 37 (84.1) | 1.98 (0.65, 6.04) |
| BCT 8. Information about others approval (off) | 11 (28.2) | 28 (71.8) | 1.00 |
| BCT 8. Information about others approval (on) | 5 (13.2) | 33 (86.8) | 2.59 (0.80, 8.36) |

| **ESM Table 13.** Effectiveness of BCTs to modify target constructs: mean scores and unadjusted and adjusted Odds Ratios – results from the univariate and multivariate binary logistic regression subgroup analyses for ethnic minority groups (n=78) | | | | | | | | | | | | | | | | |
| --- | --- | --- | --- | --- | --- | --- | --- | --- | --- | --- | --- | --- | --- | --- | --- | --- |
|  | Avoidance | | Lack of trust in Western Medicine | | Shared decision making and family influenced participation | | Colonoscopy, colon and rectum ‘culturally taboo’ topics | | Shame and embarrassment | | Fear of pain and discomfort | | Transport / travel | | Reliance on medical professional / authority | |
|  | **Mean**  **(SD)** | ***t***  **(P)** | **Mean**  **(SD)** | ***t***  **(P)** | **Mean**  **(SD)** | ***t***  **(P)** | **Mean**  **(SD)** | ***t***  **(P)** | **Mean**  **(SD)** | ***t***  **(P)** | **Mean**  **(SD)** | ***t***  **(P)** | **Mean**  **(SD)** | ***t***  **(P)** | **Mean**  **(SD)** | ***t***  **(P)** |
| BCT 1 (off) | 1.64 (0.87) | 0.342  (0.367) | - | - | - | - | - | - | - | - | - | - | - | - | - | - |
| BCT 1 (on) | 1.57  (0.94) |  | - | - | - | - | - | - | - | - | - | - | - | - | - | - |
| BCT 2 (off) | - | - | 2.00  (0.97) | 0.532  (0.596) | - | - | - | - | - | - | - | - | - | - | - | - |
| BCT 2 (on) | - | - | 1.87  (1.19) |  | - | - | - | - | - | - | - | - | - | - | - | - |
| BCT 3 (off) | - | - | - | - | 1.58  (9.70) | -1.014  (0.314) | - | - | - | - | - | - | - | - | - | - |
| BCT 3 (on) | - | - | - | - | 1.76  (0.89) |  | - | - | - | - | - | - | - | - | - | - |
| BCT 4 (off) | - | - | - | - | - | - | 2.23  (1.20) | 0.987  (0.327) | - | - | - | - | - | - | - | - |
| BCT 4 (on) | - | - | - | - | - | - | 2.02  (0.97) |  | - | - | - | - | - | - | - | - |
| BCT 5 (off) | - | - | - | - | - | - | - | - | 1.74  (0.20) | -0.790  (0.432) | - | - | - | - | - | - |
| BCT 5 (on) | - | - | - | - | - | - | - | - | 1.93  (1.02) |  | - | - | - | - | - | - |
| BCT 6 (off) | - | - | - | - | - | - | - | - | - | - | 3.35  (1.11) | -0.355  (0.723) | - | - | - | - |
| BCT 6 (on) | - | - | - | - | - | - | - | - | - | - | 3.44  (1.16) |  | - | - | - | - |
| BCT 7 (off) | - | - | - | - | - | - | - | - | - | - | - | - | 1.82  (0.98) | 0.000  (1.00) | - | - |
| BCT 7 (on) | - | - | - | - | - | - | - | - | - | - | - | - | 1.82  (0.95) |  | - | - |
| BCT 8 (off) | - | - | - | - | - | - | - | - | - | - | - | - | - | - | 1.97  (1.01) | 0.548  (0.585) |
| BCT 8 (on) | - | - | - | - | - | - | - | - | - | - | - | - | - | - | 1.84  (1.10) |  |

| **ESM Table 14.** Interactions of BCTs to modify intentions: number, proportions and unadjusted and adjusted Odds Ratios – results from the univariate and multivariate binary logistic regression analyses (n=1,288) | | | |
| --- | --- | --- | --- |
|  | **All other responses**  **(Range 1-5)** | **Yes, definitely**  **(Range 1-5)** | **OR (95%CI)** |
| BCT 1 and 5. (off) | 145 (14.3) | 866 (85.7) | 1.00 |
| BCT 1 and 5. (on) | 39 (11.7) | 293 (88.3) | 1.26 (0.86, 1.85) |
| BCT 1 and 6. (off) | 147 (14.4) | 871 (85.6) | 1.00 |
| BCT 1 and 6. (on) | 38 (11.7) | 288 (88.3) | 1.28 (0.87, 1.87) |
| BCT 5 and 6. (off) | 149 (14.5) | 879 (85.5) | 1.00 |
| BCT 5 and 6. (on) | 36 (11.4) | 280 (88.6) | 1.32 (0.89, 1.94) |
| BCT 1, 5 and 6. (off) | 169 (14.2) | 1,017 (85.8) | 1.00 |
| BCT 1, 5 and 6. (on) | 16 (10.1) | 142 (89.9) | 1.48 (0.86, 2.54) |
| BCT 3 and 4. (off) | 151 (15.0) | 859 (85.0) | 1.00 |
| BCT 3 and 4. (on) | 34 (10.2) | 300 (89.8) | **1.55 (1.05, 2.30)*** |
| BCT 3 and 7. (off) | 138 (13.7) | 866 (86.3) | 1.00 |
| BCT 3 and 7. (on) | 47 (13.8) | 293 (86.2) | 0.99 (0.70, 1.42) |
| BCT 4 and 7. (off) | 138 (13.6) | 875 (86.4) | 1.00 |
| BCT 4 and 7. (on) | 47 (14.2) | 284 (85.8) | 0.95 (0.67, 1.36) |
| BCT 3, 4 and 7. (off) | 167 (14.1) | 1,015 (85.9) | 1.00 |
| BCT 3, 4 and 7. (on) | 18 (11.1) | 144 (88.9) | 1.32 (0.79, 2.21) |

| **ESM Table 15.** Interactions of BCTs to modify target constructs: mean scores and unadjusted and adjusted Odds Ratios – results from the univariate and multivariate binary logistic regression analyses (n=1,288) | | | | | | | | | | | | | | | | |
| --- | --- | --- | --- | --- | --- | --- | --- | --- | --- | --- | --- | --- | --- | --- | --- | --- |
|  | Avoidance | | Lack of trust in Western Medicine | | Shared decision making and family influenced participation | | Colonoscopy, colon and rectum ‘culturally taboo’ topics | | Shame and embarrassment | | Fear of pain and discomfort | | Transport / travel | | Reliance on medical professional / authority | |
|  | **Mean**  **(SD)** | ***t***  **(P)** | **Mean**  **(SD)** | ***t***  **(P)** | **Mean**  **(SD)** | ***t***  **(P)** | **Mean**  **(SD)** | ***t***  **(P)** | **Mean**  **(SD)** | ***t***  **(P)** | **Mean**  **(SD)** | ***t***  **(P)** | **Mean**  **(SD)** | ***t***  **(P)** | **Mean**  **(SD)** | ***t***  **(P)** |
| BCT 1 and 5 (off) | 1.43  (0.81) | -0.799  (0.424) | - | - | - | - | - | - | 2.01  (1.15) | 0.351  (0.726) | - | - | - | - | - | - |
| BCT 1 and 5 (on) | 1.48  (0.87) |  | - | - | - | - | - | - | 1.98  (1.05) |  | - | - | - | - | - | - |
| BCT 1 and 6 (off). | 1.44  (0.82) | -0.150  (0.440) | - | - | - | - | - | - | - | - | 3.60  (1.07) | -0.119  (0.905) | - | - | - | - |
| BCT 1 and 6 (on). | 1.45  (0.82) |  | - | - | - | - | - | - | - | - | 3.60  (1.04) |  | - | - | - | - |
| BCT 5 and 6 (off) | - | - | - | - | - | - | - | - | 2.01  (1.15) | 0.269  (0.788) | **3.63**  **(1.06)** | **2.127**  **(0.034)** | - | - | - | - |
| BCT 5 and 6 (on) | - | - | - | - | - | - | - | - | 1.99  (1.06) |  | **3.49**  **(1.07)** |  | - | - | - | - |
| BCT 1, 5 and 6 (off) | 4.81  (0.55) | -1.618  (0.106) | - | - | - | - | - | - | 2.00  (1.13) | -0.199  (0.842) | 3.61  (1.06) | 0.999  (0.318) | - | - | - | - |
| BCT 1, 5 and 6 (on) | 4.88  (0.397) |  | - | - | - | - | - | - | 2.02 (1.07) |  | 3.52  (1.05) |  | - | - | - | - |
| BCT 3 and 4 (off) |  |  |  |  | 1.50  (0.72) | -1.952  (0.051) | 1.89  (0.93) | 0.510  (0.610) |  |  |  |  |  |  |  |  |
| BCT 3 and 4 (on) |  |  |  |  | 1.60  (0.89) |  | 1.86  (0.91) |  |  |  |  |  |  |  |  |  |
| BCT 3 and 7 (off) |  |  |  |  | 1.51  (0.74) | -1.102  (0.135) |  |  |  |  |  |  | 1.72  (0.94) | 0.107  (0.915) |  |  |
| BCT 3 and 7 (on) |  |  |  |  | 1.57  (0.82) |  |  |  |  |  |  |  | 1.71  (0.92) |  |  |  |
| BCT 4 and 7 (off) |  |  |  |  | 1.53  (0.76) | 0.403  (0.687) |  |  |  |  |  |  | 1.72  (0.94) | 0.417  (0.677) |  |  |
| BCT 4 and 7 (on) |  |  |  |  | 1.51  (0.77) |  |  |  |  |  |  |  | 1.69  (0.95) |  |  |  |
| BCT 3, 4 and 7 (off) |  |  |  |  | 1.52  (0.75) | -0.815  (0.415) | 1.88  (0.92) | -0.647  (0.517) |  |  |  |  | 1.73  (0.95) | 1.662  (0.097) |  |  |
| BCT 3, 4 and 7 (on) |  |  |  |  | 1.57  (0.86) |  | 1.93  (0.99) |  |  |  |  |  | 1.60  (0.85) |  |  |  |
